# Methylation in MAD1L1 is associated with the severity of suicide attempt and phenotypes of depression

**DOI:** 10.1186/s13148-022-01394-5

**Published:** 2023-01-04

**Authors:** Aleksandr V. Sokolov, Diana-Maria Manu, Didi O. T. Nordberg, Adrian D. E. Boström, Jussi Jokinen, Helgi B. Schiöth

**Affiliations:** 1 Department of Surgical Sciences, Functional Pharmacology and Neuroscience, Uppsala University, Uppsala, Sweden; 2 Department of Clinical Sciences/Psychiatry, Umeå University, Umeå, Sweden; 3 Department of Women’s and Children’s Health/Neuropediatrics, Karolinska Institutet, Stockholm, Sweden; 4 Department of Clinical Neuroscience, Karolinska Institute, Stockholm, Sweden

**Keywords:** MAD1L1, DNA methylation, Depression, Suicide

## Abstract

Depression is a multifactorial disorder representing a significant public health burden. Previous studies have linked multiple single nucleotide polymorphisms with depressive phenotypes and suicidal behavior. MAD1L1 is a mitosis metaphase checkpoint protein that has been linked to depression in GWAS. Using a longitudinal EWAS approach in an adolescent cohort at two time points (*n* = 216 and *n* = 154), we identified differentially methylated sites that were associated with depression-related genetic variants in MAD1L1. Three methylation loci (cg02825527, cg18302629, and cg19624444) were consistently hypomethylated in the minor allele carriers, being cross-dependent on several SNPs. We further investigated whether DNA methylation at these CpGs is associated with depressive psychiatric phenotypes in independent cohorts. The first site (cg02825527) was hypomethylated in blood (exp(*β*) = 84.521, *p* value ~ 0.003) in participants with severe suicide attempts (*n* = 88). The same locus showed increased methylation in glial cells (exp(*β*) = 0.041, *p* value ~ 0.004) in the validation cohort, involving 29 depressed patients and 29 controls, and showed a trend for association with suicide (*n* = 40, *p* value ~ 0.089) and trend for association with depression treatment (*n* = 377, *p* value ~ 0.075). The second CpG (cg18302629) was significantly hypomethylated in depressed participants (exp(*β*) = 56.374, *p* value ~ 0.023) in glial cells, but did not show associations in the discovery cohorts. The last methylation site (cg19624444) was hypomethylated in the whole blood of severe suicide attempters; however, this association was at the borderline for statistical significance (*p* value ~ 0.061). This locus, however, showed a strong association with depression treatment in the validation cohort (exp(*β*) = 2.237, *p* value ~ 0.003) with 377 participants. The direction of associations between psychiatric phenotypes appeared to be different in the whole blood in comparison with brain samples for cg02825527 and cg19624444. The association analysis between methylation at cg18302629 and cg19624444 and MAD1L1 transcript levels in CD^14+^ cells shows a potential link between methylation at these CpGs and MAD1L1 expression. This study suggests evidence that methylation at *MAD1L1* is important for psychiatric health as supported by several independent cohorts.

**Supplementary Information:**

The online version contains supplementary material available at 10.1186/s13148-022-01394-5.

## Introduction

Major depressive disorder (MDD) is a multifactorial disease affecting about 6% of the adult population worldwide [1]. The underlying mechanism of MDD is complex and assumed to be related to several social and neurobiological determinants, which include an individual’s genetic and epigenetic profiles, environmental factors, neuroendocrine factors, neurotransmitter biology, and structural brain alterations [1]. The diversity of symptoms in MDD is abundant, but the most common ones (which are used by DSM-5) include depressed mood, decreased pleasure related to almost any activities, weight loss, sleep issues, and others. Depending on the severity of the condition, a depressed individual might demonstrate recurrent thoughts of death and even suicidal ideation (or suicide attempts) [1].

Suicide, in turn, is another considerable public health problem. World Mental Health (WMH) Survey Initiative reported that a global lifetime prevalence of suicide attempts could reach up to 2.7% based on the data from 17 countries [2]. Similar to depression, suicidal behavior depends on several factors including social environment, biological determinants, and their interactions. Suicidal behavior is common in most severe psychiatric conditions like MDD, bipolar disorder, and schizophrenia [3]. Suicidal behavior and MDD frequently share underlying biological mechanisms, and thus many studies have identified common genetic and epigenetic alterations between the two conditions [1, 3]. For instance, the presence of genetic variants within the FKBP prolyl isomerase 5 (*FKBP5*, also known as *FKBP51*), a gene encoding enzyme related to the functioning of steroid hormone receptors [4], has been found associated with suicide attempts and depression-related Beck Depression Inventory (BDI) score [5, 6].

In addition to genetic factors, DNA methylation is one of the most intensively studied areas of epigenetic alterations in depression or increased suicide risk [7]. DNA methylation plays a role in neural biology, where methylation patterns temporarily change after neural activation [8]. Many research groups have focused on investigating associations between DNA methylation and depression/suicide [7]. Although most of the studies investigating psychiatric outcomes used whole blood DNA methylation [9], it should be kept in mind that DNA methylation is cell- and tissue-specific [10] and the correlation between the blood and brain tissue regarding DNA methylation is usually modest [11–13]. Importantly, genetic variants may interact with epigenetic shifts, and thus the effects of depression-related gene variants could be partially explained through changes in methylation [14].

Mitotic arrest deficient 1 like 1 (MAD1L1) is a component of the mitotic spindle assembly checkpoint localized on kinetochores. This protein functions to prevent the transition to anaphase of mitosis by inhibiting the anaphase-promoting complex until chromosomes are properly aligned and all kinetochores are attached to microtubules during the metaphase [15, 16]. Several studies reported that spindle and KT associated 2 (*SKA2),* a gene involved in the inhibition of the mitotic spindle assembly checkpoint complex [17], is associated with suicide and suicidal ideation [18–21]. Therefore, the checkpoint complex could be a promising candidate for association with similar phenotypes. Among several gene candidates that included components of the checkpoint [22], MAD1L1 has been reported to be associated with psychiatric outcomes. Genome-wide association studies (GWAS) and their replications have identified genetic variants of MAD1L1 that are linked to schizophrenia [23–29], bipolar disorder [29–33], and depressive phenotypes [34–38]. Several studies identified genetic loci that showed associations with more than one psychiatric condition mentioned above [39, 40]. The functional impact of identified SNPs, however, has not been studied yet.

**Fig. 1:**
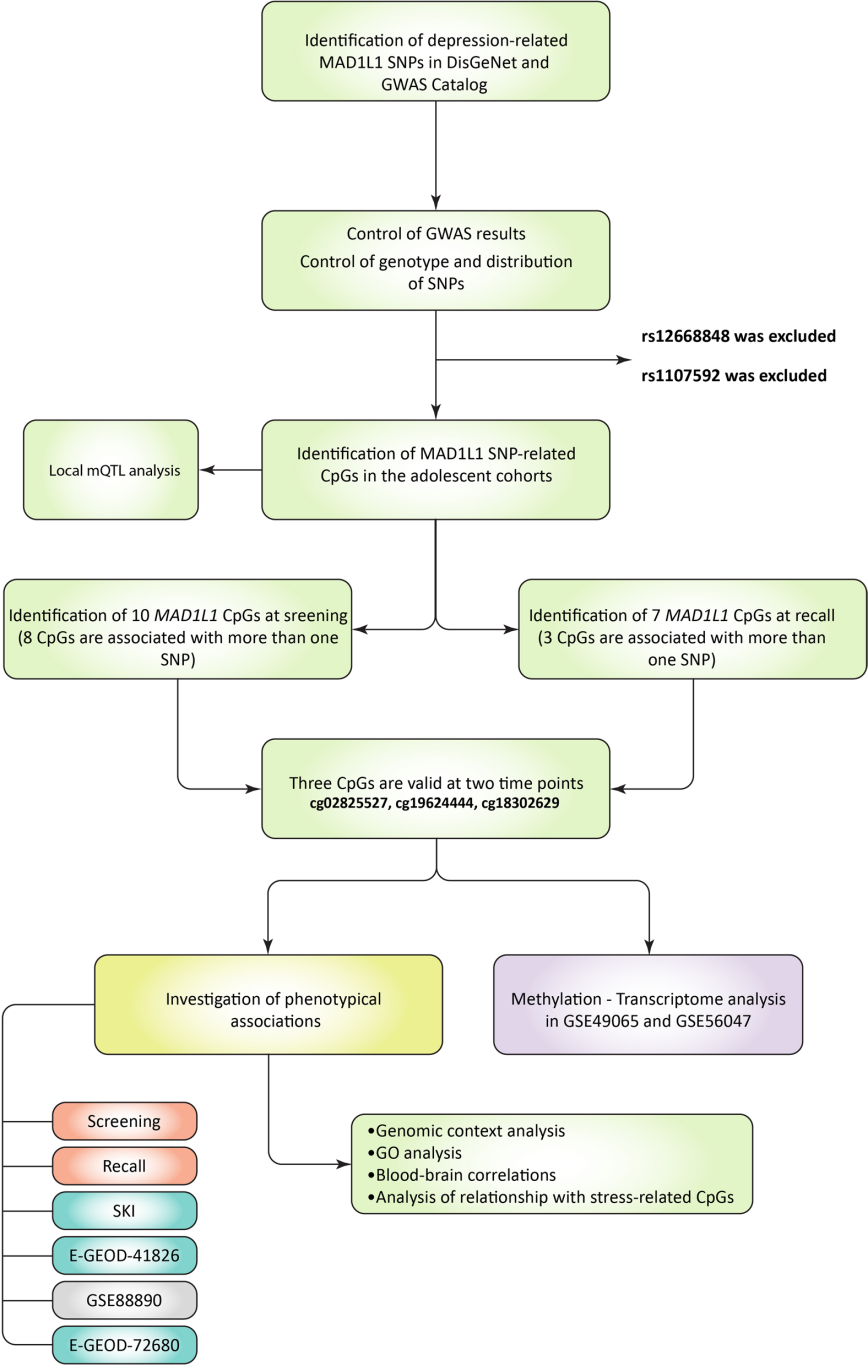
Flowchart of the analysis. This figure depicts a sequence of analytical steps performed in the current article. For detailed information regarding each step, please refer to Materials and Methods. Colors in the phenotypical analyses state the following: red—no associations were found, green—at least one of the target CpGs has shown a statistically significant association with a depressive/suicide phenotype, gray—a clear statistical tendency that failed to become statistically significant. MAD1L1, mitotic arrest deficient 1 like 1; SNP, single nucleotide polymorphism; GWAS, genome-wide association study; mQTL, methylation quantitative trait loci; GO, gene ontology

In the context of this work, we investigated how previously discovered depression-related single nucleotide polymorphisms (SNPs) at *MAD1L1* could mediate their effect through local epigenome-wide significant alterations in DNA methylation. In our analysis using two cohorts of adolescents, we applied a longitudinal epigenome-wide approach to identify blood methylation changes that are consistently associated with depressive SNPs. Furthermore, we investigated how these dependent methylation changes might be associated with the psychiatric health of individuals, including depression and suicidal behavior in several independent cohorts. Here, we report a novel methylation locus (cg02825527) that showed an association with severe suicide attempt in the blood samples. We validated this site in the analysis of postmortem brain samples of MDD individuals compared with controls. Furthermore, in two other open-access cohorts, this locus demonstrated a clear statistical tendency toward an association with similar phenotypes. We also confirm the association between cg19624444 and depression that has been identified previously [41]. Additionally, we investigated if the discovered CpG sites could be related to the expression of MAD1L1 and stress response. The workflow of the analysis is depicted in Fig. 1.

## Materials and methods

### Adolescent cohorts

To discover SNP–CpG associations, we used two adolescent cohorts: “screening” and “recall.” The study initially consisted of 786 adolescents (more than 900 as of 2022) aged 14–16 years that were randomly selected from public schools in Uppsala County, Sweden, starting from 2012. The aim of the study was to identify genetic factors and epigenetic shifts in the whole blood related to risk for psychiatric disorders among otherwise healthy adolescents. The DNA methylation analysis at the screening was conducted in two time batches: batch one—129 and batch two—92 (total 221 individuals). The recall cohort is composed of 169 individuals recruited approximately 1 year after participating in screening. Participants subject to methylation analysis in the recall were analyzed based on the depression-related scores in the computer-administered Development and Well-Being Assessment (DAWBA) questionnaire, where we selected participants with the highest and lowest scores as *cases* and *controls*, respectively (see below). Eventually, part of the recall cohort (78 adolescents) was analyzed at both screening and recall, while the other 91 individuals had DNA methylation measurement at recall only. Methylation analysis at the recall was also performed in two batches, and the batch covariable was included in the analysis.

The phenotypical assessment of individuals was undertaken during screening and recall. Height, age, and gender were self-reported, and the body weight was measured to calculate the body mass index (BMI). Participants answered a series of psychiatric health questionnaires. The computer-administered Development and Well-Being Assessment (DAWBA) questionnaire was used for two adolescent cohorts [42]. The risk for depression was categorized into six probability band scores, i.e., 0 (< 0.1%), 1 (~ 0.5%), 2 (~ 3%), 3 (~ 15%), 4 (~ 50%), and 5 (> 70%) [43]. We defined a “low-risk” group as individuals with a risk less than 50% and a “high-risk” group of individuals having a depression risk ≥ 50%.

### Suicide cohort at Karolinska Institute (SKI cohort)

Participants included in this study were invited to Suicide Prevention Clinic at the Karolinska University Hospital for the assessment of psychiatric health related to suicidal behavior and clinical follow-up. Individuals with mental retardation, schizophrenia, intravenous drug abuse, and dementia were excluded. This cohort is composed of 88 patients who at least once tried to commit suicide prior to visiting the clinic (between the years 2000–2005). A more detailed description of the cohort creation has been published previously [44]. Participants were stratified into two risk groups, i.e., severe and not severe suicide attempt. The classification criteria included either a violent suicide attempt method or a high score on the Freeman scale or eventually completed suicide (Due to January 2011). The classification of suicide violence was based on the aggressiveness of the action and the violence of the suicide method as has been proposed previously [45, 46]. Briefly, drug intake and a single wrist cut were considered as nonviolent methods, whereas everything else as violent. The Freeman scale includes two subscales—reversibility and interruption probability. The combination of scales captures the seriousness of the suicide attempt. The reversibility scale depends on the method of suicide used and on the potential damage that could be dealt by it. For instance, intoxication with a small number of pills with low toxicity is considered a reversible method, whereas self-shooting is not, and death outcome is extremely likely. The interruption probability scale, in turn, shows if the method of suicide could be interrupted by others (depending on the conditions). Each scale is graded from 1 to 5 and the combined total score—from 2 to 10 [47]. We used a cutoff score > 6 to define a serious (severe) suicide attempt. All participants were linked to the national Cause of Death register. Four of the individuals out of 88 eventually completed suicide and were included in the severe suicide group. Methylation analysis of the SKI cohort has been performed at two time points and the batch covariable was added to the model.

### Sample collection in the adolescent and SKI cohorts, genotyping and methylation profiling

Blood samples have been collected in K2EDTA blood tubes (Greiner Bio-One, Austria). Genomic DNA has been extracted using E.Z.N.A. Blood DNA Kit (Omega Bio-Tek, USA). Then, extracted DNA was used for genotyping or methylation profiling. The genotyping procedure for the initial 786 participants in the adolescent cohorts was performed using the Illumina Infinium array at the SNP&SEQ Technology Platform in Uppsala (www.genotyping.se). The facility is a part of the National Genomics Infrastructure supported by the Swedish Research Council for Infrastructures and Science for Life Laboratory, Sweden. The Illumina Infinium array includes 700,078 genetic variants. The data for other remaining SNPs were imputed using IMPUTE2 software, and prior to imputation, several quality control (QC) steps had been performed as described in our previous publication [48]. A fraction of participants were recruited after the genotyping had been performed, and thus genomic data were available for 216 out of 221 participants in the screening cohort and for 154 out of 169 in the recall cohort.

For the methylation analysis, DNA has been bisulfite-converted using the EZ DNA Methylation kit (Zymo Research, USA). All procedures have been performed according to manufacturer protocol. DNA methylation profiling was performed for the adolescent cohort at screening, using Illumina Infinium HumanMethylation450 Array. This bead chip is designed to capture ~ 450,000 methylation sites. Methylation profiling for the adolescent cohort at recall and SKI was undertaken, utilizing Illumina Infinium MethylationEPIC Array, which is designed to target ~ 850,000 CpG sites. All procedures related to sample preparations, array processing, and scanning were performed at the SNP&SEQ Technology Platform in Uppsala. For each sample, 250 ng of bisulfite-converted DNA was used.

### Open-access validation cohorts

The first open-access cohort that was used for analysis is deposited on ArrayExpress (E-GEOD-41826) and Gene Expression Omnibus (GEO)—GSE41826. The cohort includes 29 postmortem MDD samples and 29 matched controls. The prefrontal cortical samples (BA was not specified) were obtained from the NICHD Brain Bank of Developmental Disorders. Then, nuclei were extracted from cells and separated into neuronal and non-neuronal pools, using fluorescence-activated sorting, based on the expression of NeuN. A detailed description of the process is provided in the original publication [49]. The second DNA methylation cohort used in the analysis can be also found at Gene Expression Omnibus (GSE88890) and included 20 MDD suicide cases and 20 non-psychiatric sudden death controls. Tissue samples from two cortical regions (BA11, *n* = 40 and BA25, *n* = 35) were obtained from the Douglas Bell Canada Brain Bank. BA11 samples were available from all individuals, whereas three MDD and two controls for BA25 were missing. Further information could be found in the corresponding article [50]. The third validation cohort in the study is available on both ArrayExpress (E-GEOD-72680) and GEO (GSE72680). This cohort is derived from the Grady Trauma Project, a study that was conducted in Atlanta, GA, USA. Participants were characterized by multiple psychiatric risk scores, including Beck Depression Inventory [51], Childhood Trauma Questionnaire [52], and several life stress scores. Participants were also evaluated if they were under treatment for depression, anxiety, bipolar disorder, or post-traumatic stress disorder. The substance abuse was evaluated via the Kreek–McHugh–Schluger–Kellogg scale [53]. Methylation data have been obtained from blood. Further information is available in the initial publication [54].

Based on the information regarding treatment for depression, we stratified the cohort into depressed and non-depressed individuals. The initial cohort includes 422 individuals, but some of them had missing information regarding analysis-related variables: gender, age, depression treatment, BMI, and ethnicity. These individuals (45 in total) were removed from the analysis, and the resulting cohort included 377 participants.

### Data preprocessing, normalization, batch and cell-type correction, and filtering

DNA methylation data for screening, recall, and SKI cohorts were processed starting from raw IDAT files. We used a minfi-based framework [55] for data preparation. The “minfi” R package is available at bioconductor.org, a project aggregating R packages for biological applications. Signal intensities from raw files were corrected for background noise using a “noob” method [56]. Beta values were normalized, using quantile normalization, and then corrected for type I and type II probe bias via Beta Mixture Quantile Dilation [57] from the *wateRmelon* package [58]. Since different arrays in the analysis could result in batch effects, we used the “ComBat” function from the “*sva*” package to adjust for the possible bias [59, 60]. Additionally, we used a *minfi*-based implementation of the Houseman algorithm to adjust the methylation data for white peripheral blood cell heterogeneity (CD4+, CD8+, natural killer cells, B cells, monocytes, and granulocytes) [61]. Blood cell heterogeneity correction was performed at the stage of data preprocessing for adolescent cohorts and the SKI cohort. We used a regression-based approach that was described in detail previously [62], and then cell-type-corrected *M* values were applied for further analyses. Methylation data for E-GEOD-41826 were obtained with Illumina Infinium HumanMethylation450 array from DNA isolated from neuronal and non-neuronal nuclei. Quantile normalized beta values were obtained from ArrayExpress without additional normalization and adjustment procedures. Further information on the data processing is available in the initial publication [49]. Data for GSE88890 were obtained from the GEO. DNA methylation profiling for brain samples was performed with Illumina Infinium HumanMethylation450 Array. Beta values were normalized and corrected for type I and type II probe bias using similar methods as mentioned above. Similar to blood cells, different cell proportions in the brain could affect the methylation analysis as neuronal cells show different methylation profiles compared with glial cells [63]. Brain cell proportions were added to the model as covariates. We utilized a “meffil” R package to estimate the proportion of glial and neuronal cells in samples [64]. This package uses a reference methylation dataset from dorsolateral prefrontal cortex samples [49]. We performed a batch correction by including a corresponding covariable in the model. Methylation (HumanMethylation450 data) for the third validation cohort was obtained in a form of background-subtracted beta values. We used a similar procedure for normalization and probe-type bias correction as for the previous cohorts. The batch effect was corrected with the “ComBat” function. Cell proportions were estimated via a *minfi*-based Houseman algorithm [54]. Cell proportions were added to the model as covariates.

Prior to the analysis, we performed filtering and removal of the non-reliable probes from the methylation data. First, we kept the samples where more than 75% of samples have a detection *p* value less than 0.00005 and probes where more than 75% of samples have a detection *p* value less than 0.01. Probes from sex chromosomes, non-CpGs, and probes with missing beta values were removed. We removed all probes that have an SNP with a minor allele frequency (MAF) higher than 5% within the probe sequence. All probes that have an SNP at the CpG site and a single-base extension were excluded. For the HumanMethylation450 arrays, we did additional filtering, removing cross-reactive probes identified by Chen et al. [65] and Benton et al. [66], leading to 380,756 probes available at the screening analysis. For the MethylationEPIC arrays, we additionally removed cross-reactive and SNP-overlapping probes published by Pidsley et al. [67], resulting in 678,829 CpG sites at the recall analysis and 678,684 probes for SKI. In the open-access cohorts, we used only target CpG sites of interest, and all of them were presented and available after passing similar filtering steps as described above.

### Data collection and visualization

Depression-related SNPs were identified using the last updated version of the databases GWAS Catalog [68] and DisGeNet [69]. In total, eight SNPs that were associated with depression/several disorders with depression at the *MAD1L1* gene were identified. Two of the SNPs were excluded: rs12668848 since it demonstrated no confidence in association with depression [39] and rs1107592 since it has failed to reach genome-wide significance [40]. We kept rs56072378 in the analysis since it was the only SNP that was specifically associated with ICD-10-coded MDD, even though this variant did not fully reach genome-wide significance (*p* = 9*10^−7^). SNP information, such as genomic coordinates and MAF, was collected from the National Center for Biotechnology Information (NCBI) dbSNP portal (https://www.ncbi.nlm.nih.gov/snp/) [70] and from the dbSNP153 track available at the University of California, Santa Cruz (UCSC) genome browser portal (https://genome.ucsc.edu/). Linkage disequilibrium for SNPs was calculated using an online tool SNIPA with default settings [71].

Genomic context exploration was performed using the R package “gviz” [72]. Ideogram and genome axis tracks were available in *gviz*. Data for gene sequences, CpG islands, histone modifications, and transcription factor binding were obtained from the UCSC genome browser. Only depression-related transcription factors that were identified by GWAS Catalog were selected. Identification of depression-related proteins was based on the key search term “depress” applied to disease/trait description for GWAS Catalog SNPs dataset. Histone modification data deposited in UCSC were initially derived from ENCODE project [73] for normal human astrocyte (NHA) cells. Eight chromatin state tracks were obtained from Roadmap Epigenomics Project (http://www.roadmapepigenomics.org/) [74]. We obtained 15-state chromatin model data for adult brain regions (E067–E074): brain angular gyrus, brain anterior caudate, brain cingulate gyrus, brain germinal matrix, brain hippocampus middle, brain inferior temporal lobe, brain dorsolateral prefrontal cortex, and brain substantia nigra. These interactions have been generated using the multivariate hidden Markov model based on the data from 127 epigenomes [74]. For clarity, we simplified an original 15-state model to eight states. The color scheme is available in Additional files.

Spearman correlations were used to assess the correspondence between DNA methylation in blood and DNA methylation in three brain regions: BA10 (prefrontal cortex), BA20 (temporal cortex), and BA7 (parietal cortex). We used an online BECon portal to obtain the correlation data [75]. To identify the protein interaction network, we used an online database STRING that aggregates information on protein–protein associations based on experiments, database appearance of proteins, or a physical interaction [76]. We selected only physical experiment-proven interactions with a medium minimal required interaction score (0.400) as set by default. All primary interactions are shown; secondary interactions were not analyzed. For the gene ontology (GO) analysis, we used the STRING built-in tool with default settings.

### Data and statistical analysis

To investigate SNP–CpG associations, statistical analyses using R (version 4.1.1) were applied. The analysis was conducted both for screening and recall cohorts. A linear-model-based approach (*limma* package in R) applying an empirical Bayes method [77] was used. The linear model consisted of the M value quantifying the DNA methylation level as a dependent outcome and the SNP included as an independent factor. We used dominant genetic models of the SNP genotypes, i.e., minor allele is present or not present. We used a dominant model since the sample size in the study is relatively small, it simplifies the data into two categories, and it is a reason to assume that the effect of SNPs is dominant given the high rates of depression. Additionally, the use of dominant/recessive models may be beneficial in "case–control"-based designs [78]. The analysis was adjusted for sex, BMI, age, and batch covariate at screening and recall. The following formula was used for *limma*-based linear models:

$$Methylation \sim int + SNP + Sex + Age + BMI + Batch + \epsilon$$

*Limma* models obtained for each SNP site were adjusted for multiple comparisons using a false discovery rate (FDR) method. Since we tested several SNP sites for every CpG, the resulting sets of FDR-adjusted *p* values were further corrected by the number of SNP sites tested. An adjusted *p* value of < 0.05 was considered significant. It has been concluded that EWAS analyses could be prone to genomic inflation and biases [79]. We used an R package bacon to adjust the *t*-statistics from *limma* for potential bias and inflation [79]. Bias- and inflation-corrected *t*-statistics and adjusted *p* values were calculated along with standard estimations from *limma*.

We additionally performed local methylation quantitative trait loci (mQTL) analysis around the *MAD1L1* gene to investigate SNP–CpG interaction architecture using cohorts of adolescents. First, we extracted all variants and CpGs from the *MAD1L1* gene coordinates ± 10 000 bp (Chr7:1845430–2282580) that passed QC steps before imputation [48]. Second, we performed additional filtering and kept variants that had standard reference identifier “rs,” imputation info score ≥ 0.9, and A1 expected frequency between 0.1 and 0.9. Thus, 982 variants were included in the mQTL analysis. We kept only those CpG sites that passed the QC steps described in Sect. 2.5 for the adolescent screening and recall cohort. The mQTL analysis was performed with the R package “MatrixEQTL,” which uses large matrix operations to optimize the performance of such computations [80]. We used a linear model for the analysis with additive effects of investigated variants, as specified in the *MatrixEQTL* package. The analyses were adjusted for covariates that were the same as for the *limma* models above for adolescent screening and recall cohorts. Models were adjusted with the false discovery rate. To investigate linkage disequilibrium for the Top 10 impacting variants, SNIPA with default setting was applied. The R package *dplyr* was used to calculate the relative placement of CpGs and SNPs, and the *ggplot2* to plot the mQTL heatmap.

To investigate associations between methylation and psychiatric phenotype, we used a binary logistic regression model, the psychiatric phenotype was treated as a dependent binary outcome variable, i.e., individuals at low-risk vs. high-risk or individuals with severe suicidal attempt vs. non-severe suicidal attempt, and methylation M value was an independent variable. Native R-based binary logistic regression models were applied for analysis. Two-tailed *p* values for coefficients were calculated with the Wald test (implemented in R in the “summary” function). All models were adjusted for confounders depending on the particular cohort (see below). The statistical models used in the discovery cohorts were adjusted for sex, BMI, age, and batch:

$$Depression \;risk \sim int + Methylation + Sex + Age + BMI + Batch + \epsilon$$

The ethnical background was not reported in the study.

In the SKI cohort, covariates were selected based on the biological relevance, considering the following: sex, age, batch, the presence of other personality disorders (yes/no), and the status of alcohol addiction (yes/no). These models were not adjusted for BMI since these data were missing for 10 participants. The ethnical background was not included since this factor contained eight different levels, and the presence of certain groups only in cases or controls would create issues fitting a model. The final binary logistic model included:

$$Suicide\;severity \sim int + Methylation + Sex + Age + Batch + Pers.dis. + Alc.add. + \epsilon$$

In the three open-access cohorts, we used similar methods and covariables were based on biological relevance and availability of the data. We specified the model formulas for the cohorts E-GEOD-41826, GSE88890, and GSE72680, respectively:

$$\begin{aligned} & Diagnosis \sim int + Methylation + Sex + Age + Ethnicity + \epsilon \\ & Group \sim int + Methylationn + Sex + Age + Neurons + Glia + Batch.Array + \epsilon \\ & Depr.Treatment \sim int + Methylation + Sex + Age + Ethnicity + BMI + Cell.prop. + \epsilon \\ \end{aligned}$$

In the equations above, diagnosis stands for depression or control, group—suicide or control, and Depr.Treatment—treatment or no treatment, respectively. The “Cell.prop.” coefficient depicts blood cell proportion estimations for GSE72680 that were included as covariables. Given that substance misuse data were missing for many participants in GSE72680, the authors decided not to include these covariables in the analysis.

We additionally performed an exploratory analysis of associations between identified CpG candidates and stress-related DNA methylation markers that were reported previously [81–84]. We used a linear regression model (native R implementation), where methylation M value of candidate CpGs (cg02825527, cg18302629, and cg19624444) were regressed against methylation of a stress-related CpG and other covariates. We performed these analyses specifically in those cohorts where candidate CpGs were associated with the depression-related phenotype (namely SKI, GSE88890, E-GEOD-41826, and E-GEOD-72680). We used the same covariates for these models as in the analyses between methylation and a psychiatric phenotype (see above). Nominally significant associations (raw *p* value) were considered. We included only those CpG sites that passed QC steps for methylation data preprocessing (Sect. 2.5). To investigate overlaps between cohorts, an R package “visNetwork” and custom scripts were applied.

### DNA methylation–transcriptome analysis

An association analysis of identified CpG candidates with MAD1L1 transcript levels was performed based on the data from two publicly available cohorts (GSE49065 and GSE56047). The first cohort contained transcriptome and methylation data obtained from cultured peripheral blood mononuclear cells from 10 healthy male donors. The initial study investigated associations between DNA methylation and aging in the context of exposure to peroxisome proliferator WY14,643 [85]. In the present work, we included only data where cells were exposed to sham control (0.05% DMSO) to avoid potential confounding. The phenotypical data included only one additional confounder—age. The methylation profiling in GSE49065 was performed with Illumina HumanMethylation450 BeadChip, whereas transcriptome profiling was done with Affymetrix Human Gene 1.1 ST Array. For further details, please refer to the initial publication [85]. To perform the association analysis, we regressed MAD1L1 transcript levels against the methylation M values of three candidate CpG sites and adjusted the models for age.

The second cohort (GSE56047) contained a large transcriptome and methylome data from CD^14+^ samples, collected from 1202 individuals [86]. The methylation data were obtained with the Illumina HumanMethylation450 array, and transcriptome profiling was performed with Illumina HumanHT-12 V4.0 expression BeadChip. The phenotypical data included information on sex, ethnical background, age, research site, and non-targeted cell proportions. The information on sex, ethnical background, and research site is represented by the variable “RacegenderSite.” In the current work, we regressed the quantity of MAD1L1 transcripts against the methylation M value of three investigated CpG sites. The linear models were adjusted for the “RacegenderSite” covariable, age, residual cell contamination, and chip ID. We used already normalized methylation and expression data deposited at GEO in both cohorts.

## Results

### Characterization of the cohorts

The adolescent cohort was stratified into two depression risk groups: high depression risk and low depression risk. In screening, the cohort included 24 individuals in the high-risk group and 197 individuals in the low-risk group. In the recall, these numbers were as follows: high risk (*n* = 34) and low risk (*n* = 135). High-risk groups had a relatively higher proportion of women, other characteristics appeared to be similar in both screening and recall. The characteristics of the adolescent cohorts are given in Table 1.

**Table 1:** Characteristics of the adolescent cohorts

Adolescent cohorts		
DAWBA depression risk group		
	High depr. risk	Low depr. risk
*Screening*		
Participants	High depr. risk (*n* = 24 10.86%)	Low depr. risk (*n* = 197 89.14%)
Gender distribution	Men: 2 (8.3%) Women: 22 (91.7%)	Men: 54 (27.4%) Women: 143 (72.6%)
Age	15.42 ± 0.65 Min: 14, Max: 16	15.45 ± 0.63 Min: 14, Max: 17
BMI	22.51 ± 3.79 Min: 16.75, Max: 31.91	21.85 ± 3.39 Min: 15.65, Max: 37.54
*Recall*		
Participants	High depr. risk (*n* = 34, 20.12%)	Low depr. risk (*n* = 135, 79.88%)
Gender distribution	Men: 5 (14.7%) Women: 29 (85.3%)	Men: 40 (29.6%) Women: 95 (70.4%)
Age	17.71 ± 1.06 Min: 16, Max: 20	18.07 ± 0.98 Min: 15, Max: 20
BMI	23.12 ± 3.71 Min: 17.5, Max: 33.35	22.78 ± 3.14 Min: 16.27, Max: 32.02

This table shows the demographical characteristics of the adolescent cohorts in the study. Cohorts were split into subgroups based on the DAWBA depression risk group. For numerical variables, the sample mean ± standard deviation is shown. Min stands for a minimal value, and max shows the maximal value. DAWBA, Development and Well-Being Assessment; BMI, body mass index

The characteristics of the SKI cohort are given in Table 2. Thirty-one participants were classified as severe suicide attempters, whereas 57 participants were considered as nonviolent suicide attempters. Two groups showed similar profiles regarding age and BMI. The severe suicide group was very enriched with men—51% versus 21% in the nonviolent group. Most of the participants were ethnical swedes (> 65%) in both groups. Personality disorders and substance abuses were more prevalent in the violent group.

**Table 2:** Characteristics of the SKI cohort

	Severe	Not-violent
Participants	Severe	Not-violent
	(*n* = 31, 35.23%)	(*n* = 57, 64.77%)
Gender distribution	Women: 15 (48.4%)	Women: 45 (78.9%)
	Men: 16 (51.6%)	Men: 12 (21.1%)
Age	35.16 ± 12.27	33.63 ± 12.17
	Min: 19, Max: 63	Min: 18, Max: 62
BMI	24.32 ± 4.61	24.85 ± 4.31
	Min: 18.33, Max: 39.96	Min: 18.33, Max: 40.77
Ethnicity	Chilean: 0 (0%)	Chilean: 1 (1.8%)
	Finnish: 1 (3.3%)	Finnish: 5 (9.1%)
	Indian: 1 (3.3%)	Indian: 0 (0%)
	Caucasian: 1 (3.3%)	Caucasian: 0 (0%)
	Korean: 0 (0%)	Korean: 1 (1.8%)
	Mixed: 5 (16.7%)	Mixed: 12 (21.8%)
	Swedish: 21 (70%)	Swedish: 36 (65.5%)
	German: 1 (3.3%)	German: 0 (0%)
Completed suicide	Absent: 27 (87.1%)	Absent: 57 (100%)
	Present: 4 (12.9%)	Present: 0 (0%)
*Personal characteristics*		
Borderline personality disorder	Present: 7 (22.6%)	Present: 5 (8.8%)
	Absent: 24 (77.4%)	Absent: 52 (91.2%)
Other personality disorder	Present: 11 (35.5%)	Present: 10 (17.5%)
	Absent: 20 (64.5%)	Absent: 47 (82.5%)
Alcohol dependence	Present: 9 (29%)	Present: 8 (14%)
	Absent: 22 (71%)	Absent: 49 (86%)
Substance dependence	Present: 6 (19.4%)	Present: 9 (15.8%)
	Absent: 25 (80.6%)	Absent: 48 (84.2%)

This table shows the demographical characteristics of the SKI cohort in the study. The cohort was split into subgroups based on the severity of the suicide attempt. For numerical variables, the sample mean ± standard deviation is shown. Min stands for a minimal value, and max shows the maximal value. BMI, body mass index

In the cohort E-GEOD-41826, participants were presented into two groups: depressed and controls (Table 3). Both groups were identical in genders and almost identical in age and ethnical background. Individuals predominantly had Caucasian and African origin. The demographics for the GSE88890 cohort are available in the Additional file 9: Table S1. Study groups were almost identical regarding gender but appeared to be different in the ages of the participants, whereas in the suicide group, participants were on average ~ 9 years older.

**Table 3:** Characteristics of the depression cohort E-GEOD-41826

	Depression	Normal
Participants	Depression	Normal
	(*n* = 29, 50%)	(*n* = 29, 50%)
Gender distribution	Women: 15 (51.7%)	Women: 15 (51.7%)
	Men: 14 (48.3%)	Men: 14 (48.3%)
Age	32 ± 15.92	32.1 ± 16.05
	Min: 13, Max: 78	Min: 13, Max: 79
Ethnicity	African: 6 (20.7%)	African: 6 (20.7%)
	Asian: 1 (3.4%)	Asian: 0 (0%)
	Caucasian: 22 (75.9%)	Caucasian: 23 (79.3%)

This table shows the demographical characteristics of the E-GEOD-41826 depression cohort. Participants were grouped based on the depression diagnosis. For the age variable, the sample mean ± standard deviation is shown. Min stands for a minimal value, and max shows the maximal value

Demographical characteristics for the Grady Trauma Project cohort (E-GEOD-72680) are shown in Additional file 10: Table S2. The investigative sample included 377 participants after the removal of samples with missing data. Participants were from different ethnical backgrounds; however, most of them had African-American origin. Based on depression treatment, the cohort was split into two subgroups: under treatment and without treatment. These groups included 137 (36.34%) and 240 participants (63.66%), respectively. Groups appear to be different in age, BMI, and ethnicity. Also, they demonstrate different scores in almost all psychiatric tests, except for substance misuse, and many appear to have treatment for other psychiatric disorders besides depression.

### Identification of target CpGs in the adolescent cohorts

The flowchart of the analysis is presented in Fig. 1. The first step was to identify depression-related SNPs at the *MAD1L1* gene based on the previous GWAS. For this purpose, we used the GWAS Catalog and DisGeNet databases, and eight unique SNPs (rs12668848, rs56072378, rs11772627, rs3823624, rs2056477, rs11514731, rs61409925, and rs1107592) were identified as candidates for analysis. We inspected whether our adolescent cohorts at screening and recall have sufficient representation of genotypes at the specified SNPs and also whether these SNPs are indeed associated with depression or its manifestation. Two SNPs (rs12668848 and rs1107592) did not pass the control as described in the methods, and thus were excluded from the subsequent analysis. Information regarding the six remaining SNPs is available in Table 4. Subsequently, the SNP–CpG analysis applying the *limma* models included two steps, i.e., the discovery of SNP–CpGs pairs using the adolescent cohort at screening and the separate discovery of SNP–CpGs pairs at recall. Only CpGs with DNA methylation levels associated with more than one SNP were considered since the associations may provide more biological relevance. We added this restriction since we assume that the depression contribution of SNPs and their effect on methylation is likely to be shared given their close positions and association with the same gene.

**Table 4:** MAD1L1 depression-related SNPs from GWAS Catalog and DisGeNet

SNP	Chrom	Start hg19	End hg19	Ref. allele	Alt. allele	MAF	Associated gene	Uniprot ID	Functional impact
rs56072378	chr7	2104363	2104364	A	G	25 ± 5% Min: 20%, Max: 32%	MAD1L1	Q9Y6D9	Intron Variant
rs11772627	chr7	2109820	2109821	G	AC	18 ± 4% Min: 15%, Max: 27%	MAD1L1	Q9Y6D9	Intron Variant
rs3823624	chr7	2110345	2110346	T	C	18 ± 4% Min: 12%, Max: 27%	MAD1L1	Q9Y6D9	Intron Variant
rs2056477	chr7	2079743	2079744	G	ACT	28 ± 5% Min: 25%, Max: 32%	MAD1L1	Q9Y6D9	Intron Variant
rs11514731	chr7	2051502	2051503	C	G	18 ± 4% Min: 15%, Max: 27%	MAD1L1	Q9Y6D9	Intron Variant
rs61409925	chr7	1971225	1971226	G	A	18 ± 4% Min: 14%, Max: 25%	MAD1L1	Q9Y6D9	Intron Variant

This table shows the properties of the investigated SNPs. Data were obtained from the NCBI dbSNP portal. MAF is shown as a mean percent for several populations ± standard deviation of the percent. SNP, single nucleotide polymorphism; NCBI, National Center for Biotechnology Information; Chrom, chromosome; Ref, reference; Alt, alternate; MAF, minor allele frequency

**Fig. 2:**
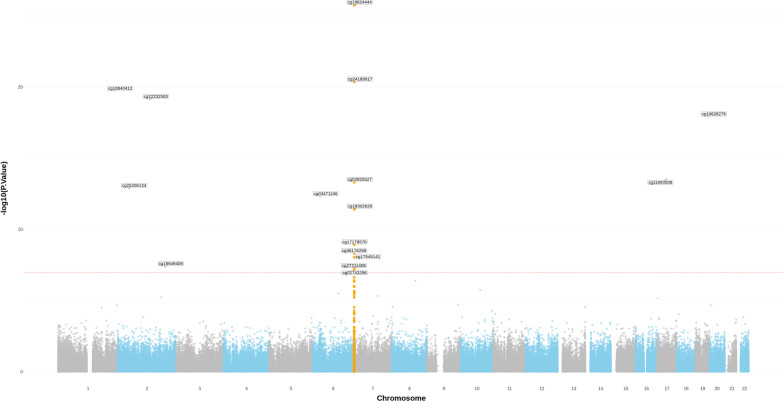
SNP-related CpGs identified at screening. This Manhattan plot shows identified SNP-related CpG sites in the screening adolescent cohort. This figure comprises results for all investigated SNP sites. All four studied SNPs were tested independently. Analyses were conducted with the R package “limma,” the dominant model was used. The smallest *p* value for every SNP–CpG association is included in the image. Raw *p* values from limma are visualized. The dashed red line shows a threshold for significance after correction for multiple testing (false discovery rate) and the number of SNP sites. Only probes that passed initial QC were included in the analysis. CpG sites located at the MAD1L1 gene are highlighted in yellow. All statistically significant results are annotated by name. SNP, single nucleotide polymorphism; QC, quality control

**Fig. 3:**
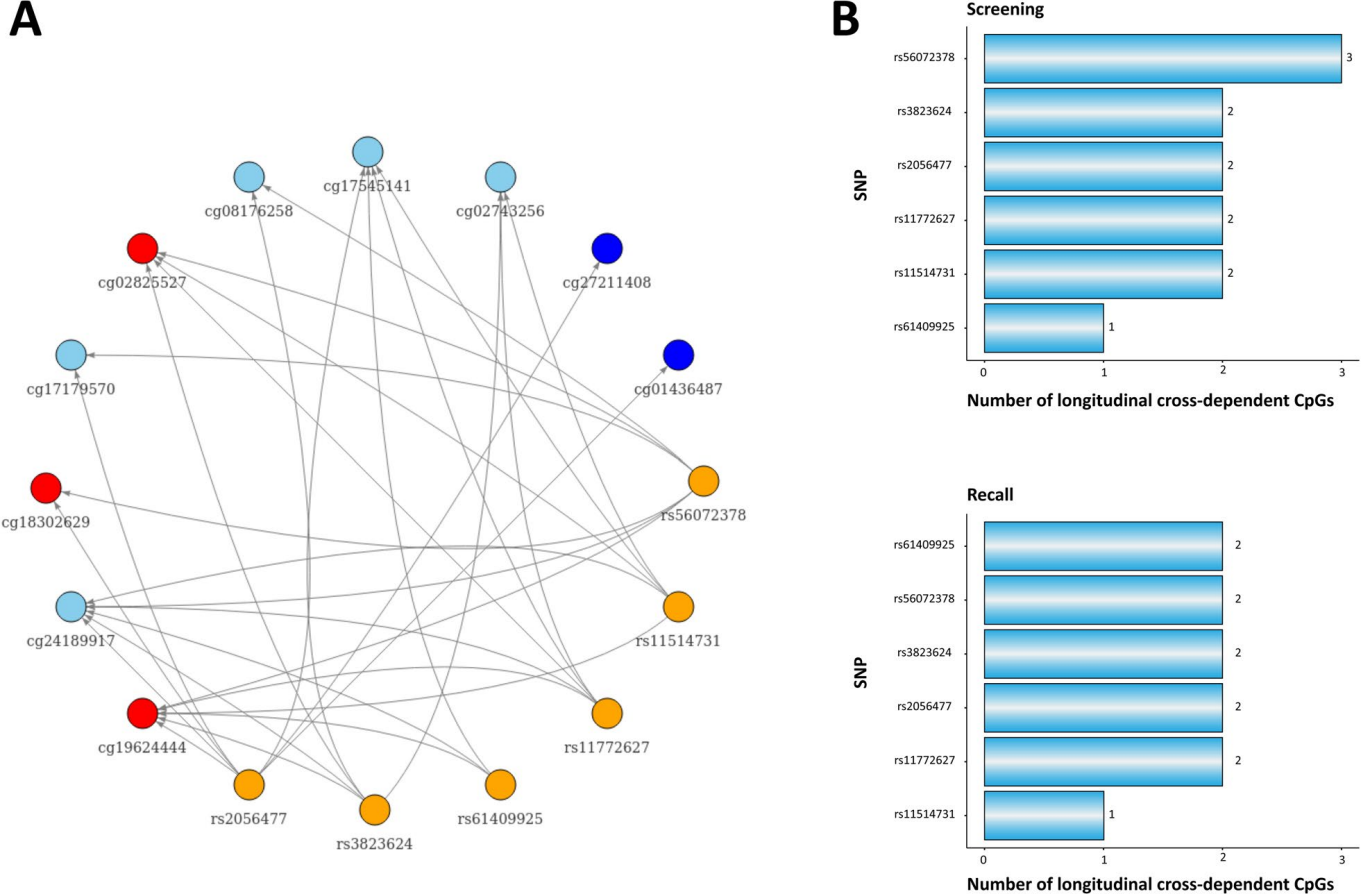
SNP–CpG associations. **A** SNP–CpG associations identified at screening adolescent cohort with limma. Nodes represent either SNP or CpG. The yellow color shows an SNP, and the blue color shows CpGs. The light blue color shows CpGs that were related to more than one SNP at screening. The red color indicates that a CpG was associated with more than one SNP both at screening and at recall. **B**. Number of longitudinal cross-dependent CpGs per SNP. This figure shows how many identified CpGs (cg02825527, cg18302629, and cg19624444) are associated with every SNP at screening and recall

The epigenome-wide analysis at screening identified 17 unique CpG sites that reached epigenome-wide significance, 10 of which were located within or nearby the *MAD1L1* gene and were associated with either of the four depression-related *MAD1L1* SNPs (Additional file 11: Table S3 and Fig. 2). Eight of these CpGs showed associations with more than one SNP site (Fig. 3A). We conducted a similar independent epigenome-wide analysis on the recall cohort and identified 18 CpGs, seven of which were located near *MAD1L1*. Three of these methylation sites (cg02825527, cg18302629, cg19624444) showed associations with more than one SNP (Additional file 11: Table S3 and Additional file 1: Fig S1), and only these CpG sites appeared both at screening and recall as cross-dependent CpGs, thus indicating that methylation changes in these CpGs are consistent and might have biological implications (Fig. 3B, C). All analyses depicted very low genomic inflation and bias (Additional file 9: Table S1) based on the estimations using the empirical null distribution [79]. Adjusting for the bias lowered the test *p* values (except for rs56072378); however, the overall results were not affected, yielding only cg02825527, cg18302629, and cg19624444 as both screening and recall cross-dependent CpGs. Identified CpGs demonstrated weak correlations at both screening and recall, with Spearman's correlation coefficients ranging from 0.21 to 0.38 (*p* values < 0.05). Only cg02825527 and cg18302629 were not correlated at screening.

Visualizations for SNP–CpG pairs for identified lead CpGs are available in Additional file 2: Fig S2 and Additional file 3: Fig S3.

At screening, genotypes at all six investigated SNPs were associated with DNA methylation levels at cg19624444, while genotypes at four SNPs were associated with DNA methylation at cg02825527, and two SNPs were associated with DNA methylation at cg18302629. Using SNIPA, linkage disequilibrium was observed between several investigated SNPs (Additional file 12: Table S4).

We additionally performed local mQTL analysis around MAD1L1 coordinates to investigate the SNP–CpG interactions in the region. These analyses were applied separately in adolescent screening and recall cohorts (Additional file 13: Tables S5.1–S5.7 and Additional file 4: Fig S4). We identified many potential SNP–CpG associations that passed adjustment for multiple comparisons. For the depression SNPs, cg19624444 was the Top 1 interacting CpG site for both screening and recall, with the exception of rs61409925:G:A at the screening where it was the second CpG. The site cg02825527 was typically around the third place, whereas cg18302629 varied from the 2-nd to 20+. Interestingly, from the CpG perspective, the depression SNPs frequently did not appear even in the Top 20 interacting SNPs regarding the FDR. However, for cg19624444, almost all Top 10 partners were in the linkage disequilibrium with depression-related MAD1L1 loci, and some of the Top 10 partners of cg02825527 were in similar LD at screening (Additional file 13: Tables S5.3 and S5.6). The heatmaps of interaction show that cg19624444 interacts with many SNPs with high effect sizes in both screening and recall samples (Additional file 5: Fig S5). Also, there are visible clusters with high effect sizes close to Chr7:1 900 000. The overall architecture of SNP–CpG interactions appears to be relatively similar at screening and recall.

### Investigation of DNA methylation at MAD1L1 gene and psychiatric outcome

**Fig. 4:**
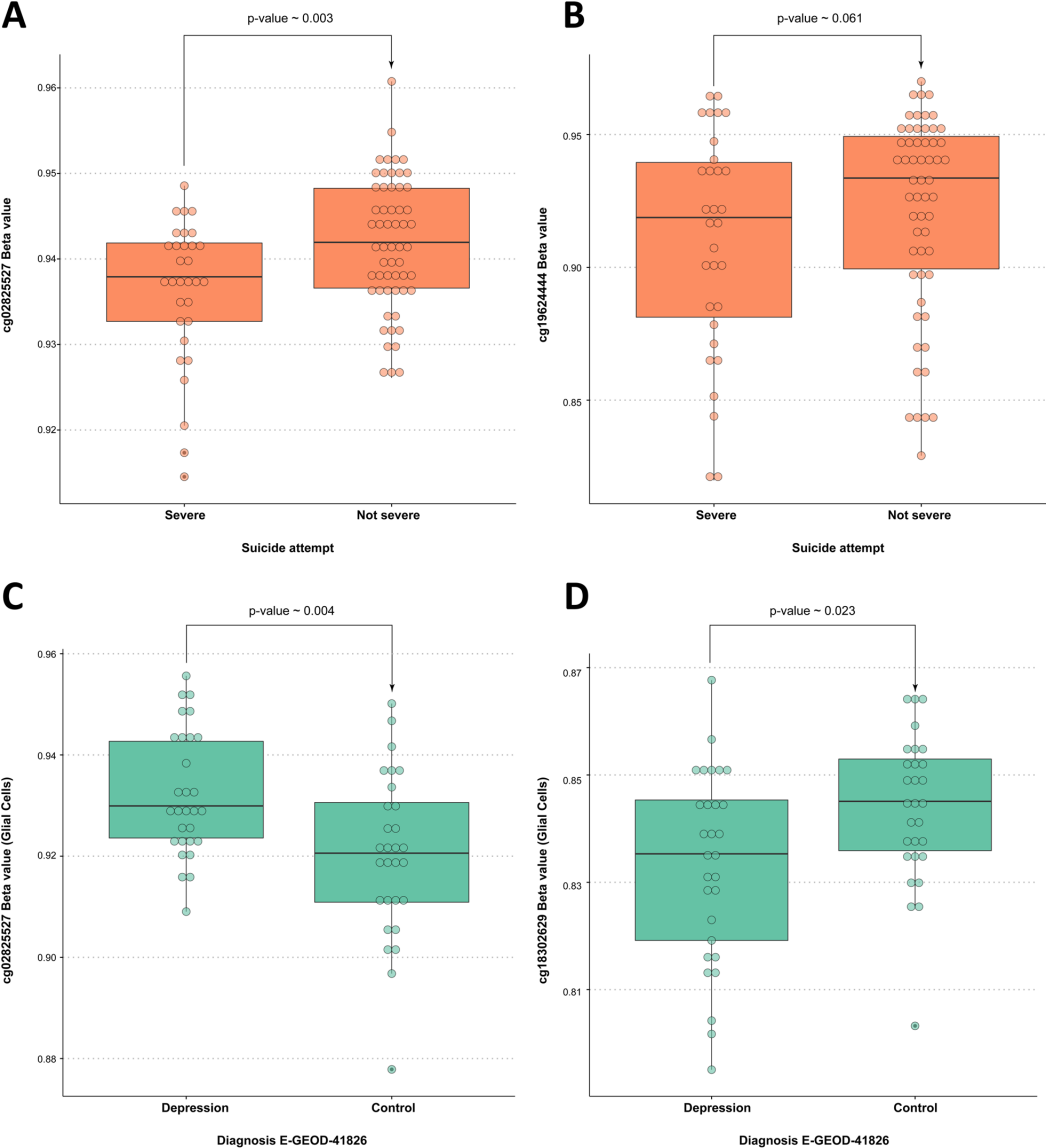
CpG–phenotype associations. **A**. Methylation at cg02825527 (*β* value) in relation to the severity of the suicide attempt in the SKI cohort. Methylation analysis is obtained from the blood sample. *p* value was derived from a binary logistic regression analysis, where methylation (*M* value) was used as a predictor and calculated using Wald statistics (implemented natively in R). **B**. Methylation at cg19624444 (*β* value) in relation to the severity of the suicide attempt in the SKI cohort. Methylation analysis is obtained from the blood sample. *p* value was derived from a binary logistic regression analysis, where methylation (*M* value) was used as a predictor and calculated using the Wald statistics (implemented natively in R). **C**. Methylation at cg02825527 (*β* value) in glial cells in relation to the diagnosis in the validation cohort E-GEOD-41826. *p* value was derived from a binary logistic regression analysis, where methylation (M value) was used as a predictor and calculated using Wald statistics (implemented natively in R)

We investigated if the candidate CpG sites demonstrate associations with psychiatric phenotypes and severity of the suicidal attempts in the discovery and SKI cohorts. Lower methylation profiles at cg02825527 were associated with the severity of the suicide attempt (exp(*β*) = 84.521, *p* value ~ 0.003) in the SKI cohort (Table 5, Fig. 4A). Similarly, DNA methylation levels at cg19624444 were lower in severe suicide attempters, though this association failed to become statistically significant (*p* value ~ 0.061, Fig. 4B) after adjustment for covariates. Adding BMI in the model results in the exclusion of 10 participants, and the results remain relatively unchanged for cg02825527 (exp(*β*) = 109.18, *p* value ~ 0.004) and cg19624444 (*p* value ~ 0.12). DNA methylation levels at none of the three CpGs were associated with the DAWBA depression band in the adolescent cohorts.

**Table 5:** Binomial logistic regression coefficients for the model with cg02825527 in the SKI cohort

Coef.	*β*	95% CI *β*	Exp(*β*)	Std. error *β*	*Z*.value	*P* value
Intercept	− 17.163	− 30.068 to − 6.213	< 0.001	6.029	− 2.847	0.004
cg02825527 *M* value	4.437	1.698 to 7.661	84.521	1.505	2.947	0.003
Sex	− 1.728	− 2.933 to − 0.626	0.178	0.582	− 2.968	0.003
Age	− 0.012	− 0.056 to 0.033	0.988	0.022	− 0.516	0.606
Batch	− 1.422	− 4.742 to 1.891	0.241	1.479	− 0.961	0.336
Pers.dis	0.649	− 0.539 to 1.833	1.914	0.599	1.084	0.279
Alc.add	0.955	− 0.362 to 2.325	2.599	0.676	1.414	0.157

This table shows coefficients for the binary logistic regression model, where the severity of a suicide attempt is a dependent outcome variable depending on methylation at cg02825527 (*M* value) adjusted for confounders. Statistics for the model were calculated using a native implementation of the binary logistic regression model in the R programming language. Confidence intervals for *β* coefficients were calculated using the R package “*stats*.” Pers.dis., personality disorders; Alc.add., alcohol addiction

### Replication of results in open-access cohorts

We sought to replicate the results in open-access cohorts. In the cohort E-GEOD-41826, we investigated our target CpGs, cg02825527, cg18302629, and cg19624444, separately in neural and glial cells. Methylation levels at cg02825527 were significantly associated with depression diagnosis (yes/no) in glial cells (exp(*β*) = 0.041, *p* value ~ 0.004, Additional file 14: Table S6) but not in neural cells. The association was in opposite direction compared with results in blood (Fig. 4C). Additionally, cg18302629 was significantly hypomethylated in glia in depressed participants in the same cohort (exp(*β*) = 56.374, *p* value ~ 0.023, Additional file 13: Table S5 and Fig. 4D). This association with depression, however, was in the opposite direction in comparison with methylation at cg02825527. Since control and suicide subgroups are identical for covariates, we also compared depressed cases and controls, using a standard *T* test (equal variance), which confirmed the results from binary logistic regression models for cg02825527 and cg18302629 (*p* value = 0.003 and 0.016, respectively). Methylation of cg19624444 did not demonstrate an association with depressive phenotypes in any cell type.

We performed an additional investigation of identified CpG sites in the second open-access cohort—GSE88890. The methylation data were available for two brain cortical regions (BA11 and BA25), and we investigated them separately. The methylation of cg02825527 in the BA11 region tended to have an association with a suicide death; however, the association failed to become statistically significant (exp(*β*) = 0.197, *p* value = 0.089, Additional file 6: Fig S6A). Importantly, the direction of the association was matching the previous results from brain tissue, i.e., suicidal attempters had higher methylation compared to non-psychiatric sudden death controls. In the BA25 region, methylation levels at cg19624444 and cg18302629 demonstrated associations with suicide (exp(*β*) = 29.755, *p* value = 0.098; exp(*β*) = 0.009, *p* value = 0.06; Additional file 6: Fig S6B and C), though without passing a significance threshold.

Lastly, we studied cg02825527, cg18302629, and cg19624444 in the Grady Trauma Project cohort (E-GEOD-72680). A binary logistic regression identified a statistical tendency toward an association between lower blood methylation at cg02825527 and treatment for depression. It should be noted that the direction of the association matches the findings from blood in the SKI cohort, although the effect size is relatively small (exp(*β*) = 1.119, *p* value ~ 0.075) and the association failed to become statistically significant. However, both groups appear to have many outliers based on box plot visualization (Additional file 6: Fig S6D). Interestingly, cg19624444, in turn, showed a strong association between lowered DNA methylation at the site and depression treatment (exp(*β*) = 2.237, *p* value ~ 0.003, Additional file 6: Fig S6E). Adding information on treatment for BD, PTSD, and anxiety in the model leads to exclusion of additional 35 participants due to missing data. Interestingly, the new model still shows a strong association for cg19624444 (exp(*β*) = 2.517, *p* value ~ 0.01); however, there was no association for two other CpGs.

### Genomic context analysis and gene ontology

**Fig. 5:**
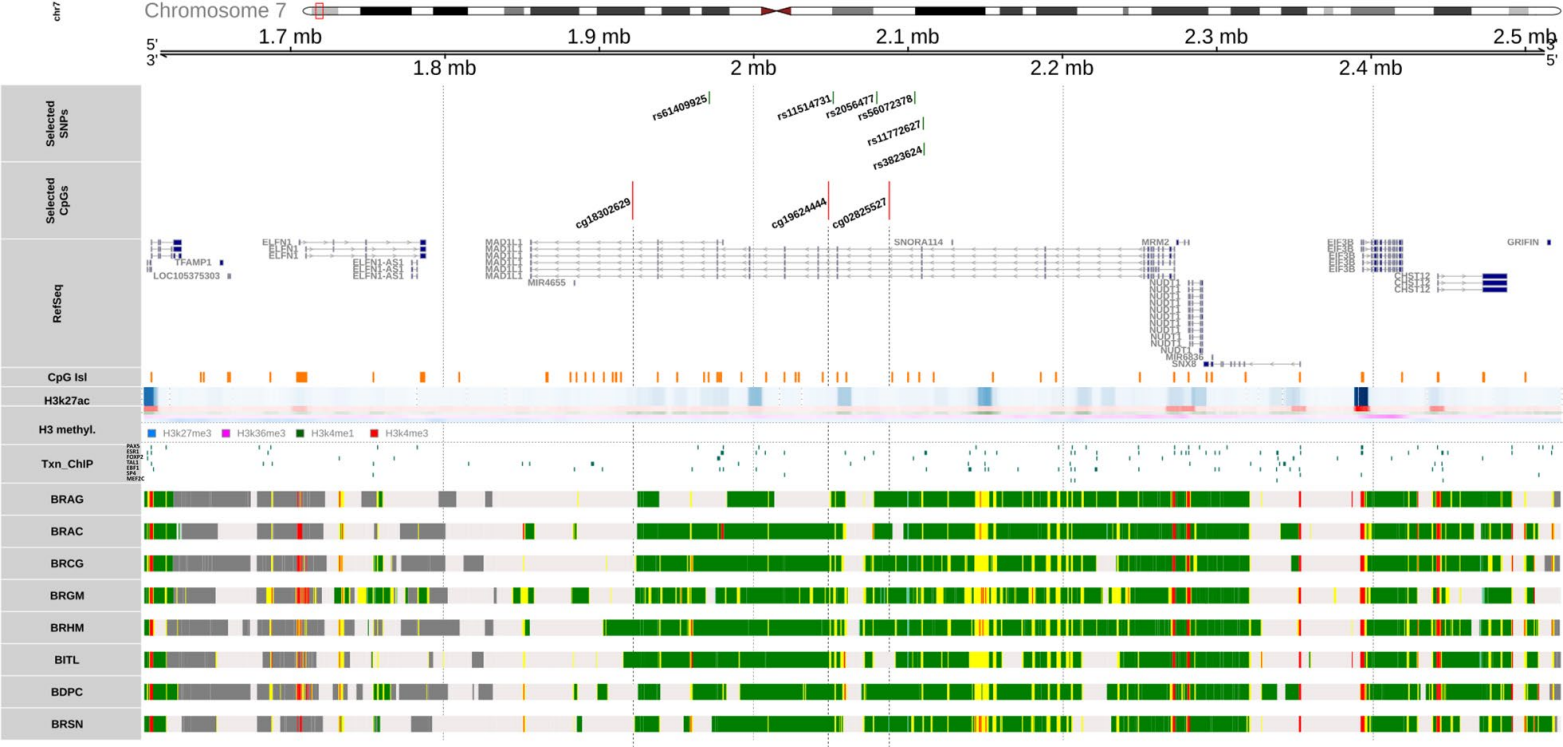
Genomic context for investigated SNPs and CpGs. This figure shows the genetic organization of the MAD1L1 gene and the location of investigated SNPs and their dependent CpGs. Ideogram and genome axis tracks were available natively in the R package “gviz.” Data for gene sequences, CpG islands, histone modifications, and transcription factor binding were obtained from the UCSC genome browser. Only depression-related transcription factors (PAX5, ESR1, FOXP2, TAL1, EBF1, SP4, and MEF2C) that were identified using GWAS Catalog were selected. For further information please refer to “materials and methods.” RefSeq track shows neighboring genes. CpGIsl track, H3k27ac, H3 methyl, and Txn_ChIP show CpG islands, histone H3 acetylation, histone H3 methylation, and transcription factor binding, respectively. The last eight tracks (BRAG to BRSN) show chromatin states for the investigated gene region. For color codes, please refer to Additional file 18: Table S10. SNP, single nucleotide polymorphism; TFAMP1, transcription factor A, mitochondrial pseudogene 1; ELFN1, extracellular leucine-rich repeat and fibronectin type III domain containing 1; ELFN1-AS1, ELFN1 antisense RNA 1; MAD1L1, mitotic arrest deficient 1 like 1; SNORA114, small nucleolar RNA, H/ACA Box 114; LOC105375303, homo sapiens uncharacterized LOC105375303; MIR4655, microRNA 4655; MRM2, mitochondrial RRNA methyltransferase 2; NUDT1, nudix hydrolase 1; MIR6836, microRNA 6836; SNX8, sorting nexin 8; EIF3B, eukaryotic translation initiation factor 3 subunit B; CHST12, carbohydrate sulfotransferase 12; GRIFIN, galectin-related inter-fiber protein; PAX5, paired box 5; ESR1, estrogen receptor 1; FOXP2, forkhead box P2; TAL1, T-cell acute lymphocytic leukemia protein 1; EBF1, early b cell factor 1; MEF2C, myocyte enhancer factor 2C; SP4, sp4 transcription factor; BRAG, brain angular gyrus; BRAC, brain anterior caudate; BRCG, brain cingulate gyrus; BRGM, brain germinal matrix; BRHM, brain hippocampus middle; BITL, brain inferior temporal lobe; BDPC, brain dorsolateral prefrontal cortex; BRSN, brain substantia nigra

We explored the spatial organization of investigated SNPs and CpGs in the genome (Fig. 5). All studied sites are located within intronic sequences of the MAD1L1 gene and the majority of investigated SNPs group closer to cg02825527 and cg19624444 compared to cg18302629. Both cg02825527 and cg19624444 are located outside of CpG Islands within the 2–4 kb range. The latter CpG cg18302629 is not located close to any CpG Island. Additionally, methylation sites at cg02825527 and cg19624444 do not overlap any histone acetylation/methylation marks, whereas cg18302629 shows overlap with increased acetylation based on the astrocyte cell data from ENCODE. Chromatin state inspection shows that cg19624444 is located in close proximity to enhancer regions in several brain tissues, particularly in the brain cingulate gyrus, brain germinal matrix, brain hippocampus middle, and brain inferior temporal lobe and is also potentially close to H3K27ac mark.

**Fig. 6:**
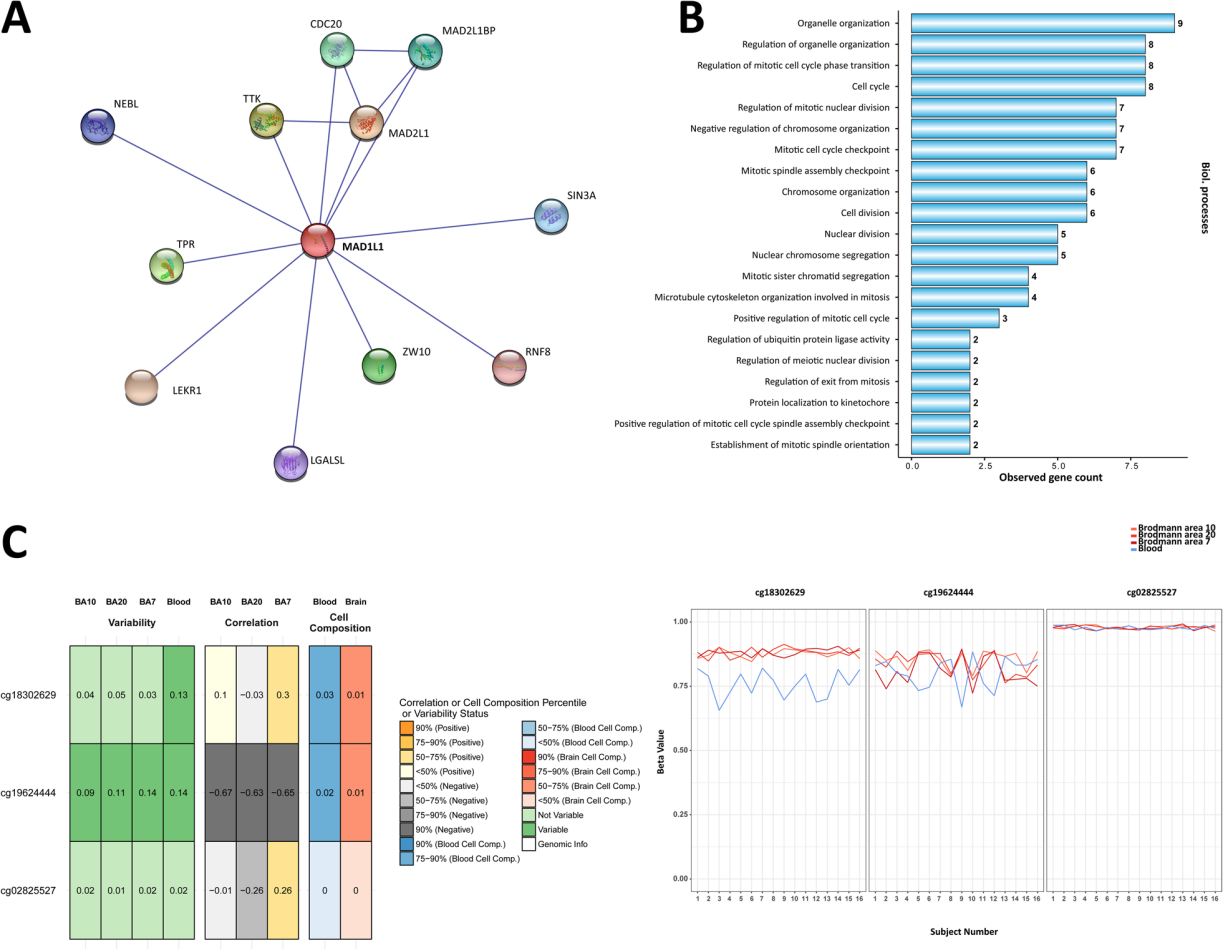
Interactors, gene ontology, and methylation blood–brain correlation. **A**. Interacting partners of MAD1L1. Physical interactions of the MAD1L1 protein are shown. Data were downloaded from the STRING database. Only physical experimental data are shown, the medium confidence score was used. **B**. Gene ontology (biological processes) for the network in sub-figure A. The graph shows biological processes and the number of genes for the processes identified in the network. **C**. Methylation blood–brain correlation for cg18302629, cg02825527 and cg19624444. This section shows Spearman correlation values between blood and a brain region. Images were obtained from BECon. Data for all sub-figures are due March 2022. MAD1L1, mitotic arrest deficient 1 like 1; CDC20, cell division cycle 20; LEKR1, leucine, glutamate and lysine-rich 1; LGALSL, galectin like; MAD2L1, mitotic arrest deficient 2 like 1; MAD2L1BP, MAD2L1 binding protein; ZW10, zw10 kinetochore protein; TPR, translocated promoter region, nuclear basket protein; TTK, TTK protein kinase; RNF8, ring finger protein 8; NEBL, nebulette; SIN3A, SIN3 transcription regulator family member A; BA, Brodmann area

The association of MAD1L1 with depression may be reflected in MAD1L1-interacting proteins and their secondary functions; thus, we have conducted an enrichment analysis for MAD1L1 physical interactors based on the data from the STRING database and its provided GO/pathway analysis tools. MAD1L1 interacts with 11 proteins based on the STRING data with a medium confidence score (Fig. 6A). These proteins, as expected, are related to the known mitosis checkpoint role of MAD1L1 as confirmed by enrichment analyses of biological processes, cellular location, molecular function, and pathways of the identified network (Fig. 6B, Additional file 15: Table S7).

### Blood–brain correlation analysis

We inspected blood–brain correlations for the identified CpG sites to explain contradictory directions of the results in the blood compared with the brain. Methylation at cg19624444 was inversely correlated in all available brain versus blood comparisons, whereas cg02825527 demonstrated a weak inverse correlation only between BA20 and blood (Fig. 6C). Methylation at cg18302629 is generally lower in blood compared with brain tissues and was in weak positive correlation with BA7. All of the investigated CpGs appear to be relatively independent of cellular compositions in the samples from blood/brain based on the BECon data.

### DNA methylation–transcriptome analysis

To explore the functional impact of identified CpGs, we performed an association analysis between methylation at targeted CpG sites and the levels of MAD1L transcripts. We performed this analysis in two publicly available cohorts (GSE49065 and GSE56047). In the first cohort, we regressed the M values of cg02825527, cg18302629, and cg19624444 against one available probe of MAD1L1 (probe 8137805, primary transcript NM_003550) and found no association for any of the CpGs (Additional file 16: Table S8). In the cohort GSE56047, however, we observed a nominally significant positive association between methylation at cg18302629 and the MAD1L1 probe ILMN_2358069 (*β* = 0.244, *p* (unadj.) = 0.01), and a trend for association between methylation at cg19624444 and intensity of the same probe (*β* = -0.124, *p* (unadj.) = 0.058). The models assume the opposite direction for both CpG sites (Table 6). No associations for cg02825527 were observed.

**Table 6:** Methylation–transcriptome analysis in the cohort GSE56047

Probe	Coef.	*β*	95% CI *β*	Std. error *β*	*T* value	*p* value	Adj. *p*
MAD1L1___ILMN_2358069	cg02825527	0.0244	− 0.1708 to 0.2195	0.0995	0.2449	0.8066	0.834
MAD1L1___ILMN_2358074	cg02825527	− 0.0059	− 0.0609 to 0.0492	0.0281	− 0.2096	0.834	0.834
MAD1L1___ILMN_2358069	cg18302629	0.2439	0.058 to 0.4298	0.0947	2.5743	0.0102	0.0611
MAD1L1___ILMN_2358074	cg18302629	− 0.0156	− 0.0682 to 0.037	0.0268	− 0.5812	0.5613	0.834
MAD1L1___ILMN_2358069	cg19624444	− 0.1242	− 0.2527 to 0.0043	0.0655	− 1.8961	0.0582	0.1747
MAD1L1___ILMN_2358074	cg19624444	− 0.0135	− 0.0498 to 0.0228	0.0185	− 0.7316	0.4646	0.834

This table shows the results for association analysis between investigated CpG sites and MAD1L1 probes in the cohort GSE56047. In the models, the transcript level was a dependent numeric outcome variable, whereas CpG and other confounders (age, “racegendersite,” residual cell contamination, and chip ids) were predictors. Statistics for the model were calculated using a native implementation of the linear regression model in the R programming language. Confidence intervals for *β* coefficients were calculated using the R package “stats.” Raw *p* values were adjusted with the false discovery rate method

### Investigation of relationship between stress-related methylation and targeted CpG sites

Given the potential association of MAD1L1 to stress [87], we performed an exploratory analysis to investigate whether methylation at identified candidate CpG sites could be systematically associated with stress-related methylation that was identified previously. To do so, we regressed methylation at cg02825527, cg18302629, and cg19624444 against methylation at previously published stress-related CpGs and covariates (see methods) in the cohorts where we observed an association (or a tendency) between methylation at any of candidate CpGs and a psychiatric phenotype. This analysis identified 54 nominally significant associations, some of which, such as cg19624444–cg06309855, cg18302629–cg05608730, and others, passed adjustment for multiple comparisons at a cohort level (Additional file 17: Tables S9.1–S9.6). We inspected nominally significant associations and how they are consistent across the samples in terms of direction. Twelve CpG pairs were observed in two samples, whereas two pairs (cg02825527–cg00130530 and cg18302629–cg00130530) were significant in three samples (Additional file 7: Fig S7). The association cg18302629–cg00130530 was consistent in three cohorts with the same positive direction (*β* = 0.237; 0.445; 0.666). In total, we identified eight nominally significant CpG pairs such as cg18302629–cg10782349 and others that had similar directions for associations (Additional file 17: Tables S9.7-S9.8).

## Discussion

This is the first study showing associations between DNA methylation and previously identified depression-related SNPs at the *MAD1L1* gene and depression and suicidal behavior using multiple independent cohorts. Previous studies identified multiple genetic variants in MAD1L1 that were associated with psychiatric disorders. Variants used in the analysis were primarily associated with depression. However, rs2056477 also showed an association with adult body height based on GWAS [88], and rs3823624 was associated with duration of sleep [89]. We identified three methylation loci that were dependent on several genetic variants and the associations remained preserved longitudinally. In this setting, consistent SNP–CpG associations may be more relevant to depression, especially if they are associated with several depression-related SNPs. The interesting aspect of the findings is that associations were consistently inverse depending on whether a sample has been derived from blood or brain tissue, thus highlighting the importance of tissue-specific methylation patterns in relation to depression. In the blood-based cohorts, the individuals at high risk for depression tended to demonstrate decreased methylation of the identified CpGs, whereas in postmortem brain samples, there was a slight increase in methylation. These associations appear to be related not only to the anatomical location of the sample source but also to the cell type. In the E-GEOD-41826 data, for instance, a clear association between MDD and cg02825527 or cg18302629 was observed only in glial cells, but not in neural ones.

We did not find any published articles on cg02825527 and cg18302629 loci in relation to depression nor for other diseases or biological processes. However, a recent study by Shen et al. identified cg19624444 as a depression-related methylation site that was associated with a depression polygenic risk score. This CpG has also shown casual associations based on Mendelian randomization analysis conducted in the same study [41]. Our SNP–CpG analysis with *limma* identified cg19624444 as a top hit based on adolescent cohorts at screening and recall using a dominant genetic model. This CpG was a Top 1 interacting partner for almost all depression-related MAD1L1 SNPs in mQTL analysis with the additive genetic model as well, and its top 10 interacting SNPs were almost always in LD with depression-related MAD1L1 loci. Our results indicate the relationship between depression and methylation at cg19624444 based on the treatment data from E-GEOD-72680. Additionally, methylation levels at this CpG were associated with the severity of suicide attempt, but our analysis has insufficient statistical power to fully confirm it. Another study identified the cg08985282 site at *MAD1L1* as a depression-related locus based on a comparison of depressed individuals with their healthy monozygotic twins (12 pairs) [90]. This probe, however, did not pass the QC in our analysis since it was found to align with more than one genome site based on the list published previously [66].

Investigated methylation sites are located within the gene body of *MAD1L1*. There is a general assumption that methylation within a promoter region of a gene tends to have a negative impact on a gene expression, leading to its silencing [91]. Methylation of the gene body, on the other hand, might be associated with increased gene expression as was shown in dividing cells, but not in nondividing cells such as brain cells [92]. This difference adds up to difficulties with interpreting results from methylation studies, and the regulatory role of gene body methylation/demethylation is still incompletely understood. The localization of CpGs provides rather weak evidence that methylation of these sites impacts the expression of *MAD1L1* or other genes. Also, we do not observe any overlap with depression-related transcription factor binding sites. However, the expression of MAD1L1 may be regulated by other transcription factors. We were able to observe a potential overlap of cg19624444 with acetylation marks; however, they are relatively distant. Interestingly, the transcriptome-methylation association analysis, in turn, indicates a potential relationship between methylation at cg18302629 and cg19624444 with expression levels of MAD1L1. However, we are not able to investigate if these associations hold in the brain tissue, and in glial cells in particular. Additionally, the opposite directions for both CpGs and the absence of associations for cg02825527 provide fairly little clarity in the interpretation given that both MAD1L1 Illumina probes in the analysis correspond to the same transcript. Additionally, MAD1L1 could be expressed in form of alternative variants, which could be affected by methylation at investigated CpGs and left undetected by the transcriptome array. However, the three candidate CpGs predominantly do not overlap with transcription start sites/promoter regions of alternative MAD1L1 variants (Additional file 8: Fig S8).

The metaphase checkpoint protein MAD1L1 is primarily responsible for the cell cycle regulation, and thus it might be important for neural function only in the early stage of the lifespan since neurons are not capable of cell division, and adult neurogenesis occurs only in a few areas of the brain [93, 94]. This could explain that the association of methylation at cg02825527 and cg18302629 with depression was noticeable only in glial cells in postmortem brain samples from E-GEOD-41826 since glial cells are capable of regeneration [95]. Some evidence suggests that depression could be associated with the function of glial cells, such as microglia and oligodendrocytes, and their disruption could be associated with the disease [96, 97]. MAD1L1 protein physically interacts with 11 partners associated with its main mitosis checkpoint function, and these proteins could be potentially related to depression as well. Though such associations have not been reported yet. As mentioned earlier, several studies identified the association between *SKA2* gene that is functionally related to MAD1L1 with suicide and suicidal ideation [18–21]. This gene is a component of the SKA complex that functions to silence spindle assembly checkpoint (which includes *MAD1L1*) to promote cell cycle progression [17]. SKA2 was reported to be associated with cortisol stress reactivity [98]. Additionally, MAD1L1 is also associated with stress responses based on several studies [87]. We were able to observe several associations between candidate CpG sites and previously reported stress-related CpG marks. Particularly, two pairs (cg02825527–cg00130530 and cg18302629–cg00130530) were observed in three samples, and notably, cg00130530 is located at *FKBP5* gene that was previously reported to be associated with suicide and depression [5, 6]. Also, for instance, the pair cg18302629–cg10782349 was observed in two samples, having a similar direction for the association. The CpG cg10782349 is located at Zinc Finger Protein 701 (*ZNF701*) and was reported to be associated with stress-related suicidal ideation [84]. Additionally, similar pairs with consistent directions cg02825527–cg06092834 and cg18302629–cg12282311 also depend on CpGs related to suicidal ideation and stress [84]. Taken together, our results with previous findings on SKA2 indicate a relationship between the function of MAD1L1 and psychiatric health and also provide small evidence (given the exploratory nature and possible statistical inflation of such analysis) that stress response may be involved in the process.

It should be noted that our study has limitations. First, individual cohorts have a relatively small number of participants, and thus results should be interpreted with caution. Additionally, the cohorts at screening and recall do not fully overlap, so conclusions regarding longitudinal changes in methylation need to be studied separately in a larger cohort. Even though the SNP–CpG interactions appear similar at screening and recall based on mQTL analysis, the differences in genetic architectures may affect SNP–CpG pairs. Also, there are potential genetic differences in other cohorts used in the study. Given that investigated CpGs show either no or inverse correlation with methylation in the brain and phenotypical associations were also inverse, the biological impact of differential methylation is not clear. The relatively weak association between methylation at investigated CpGs and MAD1L1 transcriptome in CD^14+^ cells may not be reflected in the brain. We could not draw conclusions regarding the causality of identified methylation changes given the overall cross-sectional design of the study. Additionally, participants in the adolescent cohorts in the present study were not evaluated by a psychiatrist, and thus it limits the clinical validity of associations (even though we did not observe them in these cohorts). Also, the analyses may be affected by hidden confounding factors, such as antidepressant intake. This is particularly important for the cohorts investigating suicide, given that these individuals are likely to have some kind of antidepressant treatment. The strength of our study, however, is a longitudinal approach to identify consistently differentially methylated loci coupled with the use of several independent cohorts for phenotypical associations and transcriptome analysis. Such an approach should increase the likelihood to identify biologically relevant methylation changes and could avoid potential hidden biases in comparison with a single cohort.

Our results suggest an association between methylation changes in the *MAD1L1* gene in relation to suicide severity and depression. There is weak evidence of an association between methylation at cg18302629 and cg19624444 with an expression of MAD1L1 in CD^14+^ cells. However, there are no available data if the expression levels of MAD1L1 in glia are related to depressive phenotypes or whether the investigated CpGs affect MAD1L1 expression in the brain. The logical step forward would be an investigation of genetic, epigenetic, and proteomic profiles simultaneously. Ideally, this should be studied both in the blood and in the brain samples, so a potential explanation of brain changes would also offer a path for a clinical diagnostic/screening application.

## Supplementary Information

**Additional file 1: Fig S1.** This Manhattan plot shows identified SNP-related CpG sites in the recall adolescent cohort. This figure comprises results for all investigated SNP sites. All four studied SNPs were tested independently. Analyses were conducted with the R package “limma,” the dominant model was used. The smallest p value for every SNP–CpG association is included in the image. Raw p values from limma are visualized. The dashed red line shows a threshold for significance after correction for multiple testing (false discovery rate) and the number of SNP sites. Only probes that passed the initial QC were included in the analysis. CpG sites located at the MAD1L1 gene are highlighted in yellow. All statistically significant results are annotated by name. Abbreviations: SNP, single nucleotide polymorphism; QC, quality control.

**Additional file 2: Fig S2.** This figure shows the DNA methylation (β value) of identified lead CpGs in relation to alleles of all related investigated SNPs based on data at screening. All depicted associations were found to be statistically significant after correction for multiple testing and the number of SNP sites using *limma-*based models. For rs2056477, there were 2 alternative alleles available, and all discovered different genotypes are depicted.

**Additional file 3: Fig S3.** This figure shows the DNA methylation (β value) of identified lead CpGs in relation to alleles of all related investigated SNPs based on data at recall. All depicted associations were found to be statistically significant after correction for multiple testing and the number of SNP sites. For rs2056477, there were 2 alternative alleles available, and all discovered different genotypes are depicted.

**Additional file 4: Fig S4.** This figure shows QQ plots of the p-values obtained in the mQTL analysis in the adolescent screening and recall samples. The figure **A** corresponds to screening, whereas the figure **B** corresponds to recall.

**Additional file 5: Fig S5.** This figure shows heatmaps of the SNP–CpG interactions in the mQTL analysis. The X-axis shows coordinates for SNPs, the Y-axis represents coordinates for CpGs. Both coordinates correspond to the genome assembly hg19. The first row with plots shows heatmaps for -log10 p values (raw) and beta coefficients in the linear models (adjusted for age, sex, batch, and BMI) for the adolescent screening sample. The second row depicts similar maps for the adolescent recall sample. The linear models were generated with the R package *MatrixEQTL* and assumed additive genetic effects and the heatmaps were created with *ggplot2* (see methods).

**Additional file 6: Fig S6. A. Methylation of cg02825527 in BA11 brain region in the GSE88890 cohort.** This figure shows methylation of cg02825527 (β-value) for suicide and control individuals in GSE88890. **B. Methylation of cg19624444 in BA25 brain region in the GSE88890 cohort.** This figure shows methylation of cg02825527 (β-value) for suicide and control individuals in GSE88890. **C. Methylation of cg18302629 in BA25 brain region in the GSE88890 cohort.** This figure shows methylation of cg02825527 (β-value) for suicide and control individuals in GSE88890. **D. Methylation of cg02825527 in E-GEOD-72680.** This graph shows methylation of cg02825527 (β-value) for individuals on antidepressant treatment and without it. **E. Methylation of cg19624444 in E-GEOD-72680.** This figure depicts methylation of cg19624444 (β-value) for individuals on antidepressant treatment and without it.

**Additional file 7: Fig S7.** This figure shows associations between candidate CpGs and stress-related CpGs in the samples where we observed associations (or trends) between MAD1L1 methylation and depression/suicide phenotype. The figure includes all pairs (without accounting for direction) that were nominally significant based on the linear model adjusted for the cohort-specific covariates. The color of the nodes indicates the following: green—sample, blue—CpG–CpG pairs that were observed in one cohort, orange—CpG–CpG pairs that were observed in two cohorts, red—CpG–CpG pairs that were observed in more than two cohorts.

**Additional file 8: Fig S8. A.** This figure shows alternative transcripts of MAD1L1 obtained from the AceView portal (https://www.ncbi.nlm.nih.gov/IEB/Research/Acembly/av.cgi?db=human&term=MAD1L1&submit=Go) and the relative positions of candidate CpG sites. **B.** This figure shows NCBI RefSeq tracks of the *MAD1L1* gene obtained from the UCSC genome browser. The red MAD1L1 transcript is associated with Illumina HumanHT-12 V4.0 MAD1L1 probes. The orange MAD1L1 transcript is associated with the Affymetrix Human Gene 1.1 ST MAD1L1 probe.

**Additional file 9: Table S1.** Demographical characteristics of the GSE88890 cohort for two brain regions BA11. Participants were grouped based on the death cause: suicide or non-psychiatric sudden death. For the age variable, sample mean ± standard deviation is shown. Min stands for a minimal value, and max shows the maximal value.

**Additional file 10: Table S2.** Demographical characteristics of the E-GEOD-72680 cohort. Participants were grouped based on the depression treatment status. For numerical variables, sample mean ± standard deviation is shown. Min stands for a minimal value, and max shows the maximal value. Abbreviations: BMI, body mass index; BD, bipolar disorder; CTQ, Childhood Trauma Questionnaire; PTSD, post-traumatic stress disorder; BDI, Beck's Depression Inventory.

**Additional file 11: Table S3.** Results for SNP–CpG association analysis based on R limma models. Tables for screening and recall are provided separately. Limma models were run independently for every SNP site for all available CpG sites; only statistically significant results are shown. Adjusted p values were calculated using the false discovery rate method for every SNP separately. Adjusted p values were further adjusted by the number of SNP sites tested. Additionally, moderated t values from limma were adjusted for genomic inflation and bias with the R package bacon. Similar statistics as for limma t values were calculated for bacon-adjusted t values. Cumulated positions for CpGs were calculated based on the data from (https://www.ncbi.nlm.nih.gov/grc/human/data?asm=GRCh37). Associated genes and coordinates for CpGs were obtained from Illumina annotation files for a corresponding array. Column names state the following: Gene, a gene associated with an SNP; SNP, investigated SNP; SNP coords, coordinates of SNP; CpG; associated CpG site; CpG chrom., a chromosome of a CpG; CpG pos., coordinates of a CpG (for the current chromosome); CpG cumul. Pos., the cumulative position of a CpG; CpG gene, a gene associated with a CpG; logFC, estimate of the log2-fold-change corresponding to the effect; AveExpr, average log2 value for the probe over all arrays and channels; t, moderated t-statistic; P.Value, raw p value; adj.P.Value, FDR-adjusted p value with no correction for the number of SNPs and inflation/bias; B, log-odds that the site is differentially methylated; adj.P.Val.SNP, limma FDR-adjusted p value corrected for the number of SNPs; Inflation.Bacon, inflation factor calculated in bacon; Bias.Bacon, bias of estimations; T.Bacon, corrected moderated T statistics; P.val.Bacon, p value after correction for inflation and bias; Adj.P.Val.Bacon, FDR-adjusted p value after correction for inflation and bias; adj.P.Val.SNP.Bacon, FDR-adjusted p value corrected for the number of SNPs, inflation, and bias. Abbreviations: SNP, single nucleotide polymorphism; FDR, false discovery rate.

**Additional file 12: Table S4.** Linkage disequilibrium for investigated SNPs based on the output from (https://snipa.helmholtz-muenchen.de/snipa3/) with default settings. Only associations between investigated SNPs are shown. Column description provided by SNIPA: QRSID, Query SNP rsID; RSID, Proxy SNP rsID; RSALIAS, Proxy SNP alias rsID(s); CHR, Chromosome; POS1, Sentinel SNP Position; POS2, Proxy SNP Position; DIST, Distance; R2, LD r^2; D, LD D; DPRIME, LD D'; MAJOR, Proxy Allele A; MINOR, Proxy Allele B; MAF, Allele B Frequency; CMMB, Recombination Rate (CM/Mb); CM, Genetic distance (CM); COMPEFFECTS, Compressed functional annotation of Proxy SNP; TRAIT, Associated with trait (yes/no); CISEQTL, Associated with eQTL in cis (yes/no); TRANSEQTL, Associated with eQTL in trans (yes/no); GENES, Genes hit or close-by (distance 5 KB max); REGGENES, Potentially regulated genes (linked via promoter or enhancer); EQTLGENES, Genes linked via eQTL associations. Abbreviations: SNP, single nucleotide polymorphism.

**Additional file 13: Table S5. 5.1** Genetic variants used for mQTL analysis. The column headers correspond to the IMPUTE2 output. **5.2** The mQTL analysis in the adolescent screening sample. The statistics were calculated in the package *MatrixEQTL.* P values were adjusted with false discovery rate method. **5.3** Statistics of the candidate CpG sites in screening. Depr. SNP placement column indicates a relative placement of a MAD1L1 depression-related SNP in the CpG-specific mQTL table (sorted by FDR). Depr. SNP name column indicates the name of the MAD1L1 depression-related SNP in the column with placement. Top 10 Variants column shows the Top 10 variants associated with a CpG in the CpG-specific mQTL table (sorted by FDR). LD with depr.SNPs column shows if the SNP in the Top 10 list is in LD with at least one MAD1L1 depression-related SNP. **5.4** Statistics of the MAD1L1 depression-related SNPs in screening. The Top CpG placement column shows a relative placement of a candidate CpG site in the SNP-specific mQTL table (sorted by FDR). The CpG name column shows the name of the CpG associated with its placement in the previous column. **5.5** The mQTL analysis in the adolescent recall sample. The statistics were calculated in the package *MatrixEQTL.* P values were adjusted with the false discovery rate method. **5.6** Statistics of the candidate CpG sites in the recall. Depr. SNP placement column indicates a relative placement of a MAD1L1 depression-related SNP in the CpG-specific mQTL table (sorted by FDR). Depr. SNP name column indicates the name of the MAD1L1 depression-related SNP in the column with placement. Top 10 Variants column shows the Top 10 variants associated with a CpG in the CpG-specific mQTL table (sorted by FDR). LD with depr.SNPs column shows if the SNP in the Top 10 list is in LD with at least one MAD1L1 depression-related SNP. **5.7** Statistics of the MAD1L1 depression-related SNPs in the recall. The Top CpG placement column shows a relative placement of a candidate CpG site in the SNP-specific mQTL table (sorted by FDR). The CpG name column shows the name of the CpG associated with its placement in the previous column.

**Additional file 14: Table S6.** Coefficients for binary logistic regression models, where depression diagnosis is a dependent outcome variable depending on methylation at cg02825527 (M value) or cg18302629 (M value) in glial cells adjusted for confounders. Statistics for the models were calculated using a native implementation of the binary logistic regression model in the R programming language.

**Additional file 15: Table S7.** Results for gene ontology analysis for MAD1L interacting partners based on the STRING database data. For further information, please refer to the STRING database. Analysis was conducted with default settings. Abbreviations: TFAMP1, transcription factor A, mitochondrial pseudogene 1; ELFN1, extracellular leucine-rich repeat and fibronectin type III domain containing 1; ELFN1-AS1, ELFN1 antisense RNA 1; MAD1L1, mitotic arrest deficient 1 like 1; SNORA114, small nucleolar RNA, H/ACA Box 114; LOC105375303, homo sapiens uncharacterized LOC105375303; MIR4655, microRNA 4655; MRM2, mitochondrial RRNA methyltransferase 2; NUDT1, nudix hydrolase 1; MIR6836, microRNA 6836; SNX8, sorting nexin 8; EIF3B, eukaryotic translation initiation factor 3 subunit B; CHST12, carbohydrate sulfotransferase 12; GRIFIN, galectin-related inter-fiber protein; PAX5, paired box 5; EP300, E1A binding protein p300; SP4, sp4 transcription factor; BRAG, brain angular gyrus; BRAC, brain anterior caudate; BRCG, brain cingulate gyrus; BRGM, brain germinal matrix; BRHM, brain hippocampus middle; BITL, brain inferior temporal lobe; BDPC, brain dorsolateral prefrontal cortex; BRSN, brain substantia nigra.

**Additional file 16: Table S8.** Coefficients for candidate CpGs in the transcriptome–methylation association analysis in GSE49065. The expression of MAD1L1 was regressed against a candidate CpG site and age. Each row shows coefficients and estimates with statistics obtained from individual linear models. The models were implemented in R, using the standard implementation of linear regression. Confidence intervals were calculated with the R package *stats*.

**Additional file 17: Table S9. 9.1** The source publications of stress-related CpG sites. **9.2** CpG–CpG associations in the SKI. The models were calculated, using the standard implementation of linear regression. The covariates are specific for every cohort (see methods). The p values were adjusted with the false discovery rate method. The Stress CpG genes were obtained from Illumina annotation files for HumanMethylation450 and MethylationEPIC arrays. **9.3** CpG–CpG associations in the Grady sample. **9.4** CpG–CpG associations in the E-GEOD-41826 (glia) sample. **9.5** CpG–CpG associations in the GSE88890 (BA11) sample. **9.6** CpG–CpG associations in the GSE88890 (BA25) sample. **9.7** Pooled CpG–CpG associations that were nominally significant and detected more than once. **9.8** Pooled CpG–CpG associations that were nominally significant, were detected more than once, and had matching directions for beta coefficients.

**Additional file 18: Table S10.**Color codes for chromatin states tracks in Fig. 5.

## Data Availability

The datasets generated and/or analyzed in the current study are available from the corresponding author upon reasonable request.

## Abbreviations

BRAC: Brain anterior caudate
BRCG: Brain cingulate gyrus
BDPC: Brain dorsolateral prefrontal cortex
BRGM: Brain germinal matrix
BRHM: Brain hippocampus middle
BITL: Brain inferior temporal lobe
BRSN: Brain substantia nigra
BA: Brodmann area
CHST12: Carbohydrate sulfotransferase 12
CDC20: Cell division cycle 20
Chrom: Chromosome
DAWBA: Development and Well-Being Assessment
DNAm: DNA methylation
EBF1: Early b cell factor 1
ELFN1-AS1: ELFN1 antisense RNA 1
EWAS: Epigenome-wide association studies
ESR1: Estrogen receptor 1
EIF3B: Eukaryotic translation initiation factor 3 subunit B
ELFN1: Extracellular leucine-rich repeat and fibronectin type III domain containing 1
FKBP5: FKBP prolyl isomerase 5
FACS: Fluorescence-activated cell sorting
FOXP2: Forkhead box P2
GRIFIN: Galectin-related inter-fiber protein
LGALSL: Galectin like
GWAS: Genome-wide association studies
LOC105375303: Homo sapiens uncharacterized LOC105375303
LEKR1: Leucine, glutamate, and lysine-rich 1
mQTL: Methylation quantitative trait loci
MAD2L1BP: MAD2L1-binding protein
MDD: Major depressive disorder
MIR4655: MicroRNA 4655
MIR6836: MicroRNA 6836
MAF: Minor allele frequency
MRM2: Mitochondrial RRNA methyltransferase 2
MAD1L1: Mitotic arrest deficient 1 like 1
MAD2L1: Mitotic arrest deficient 2 like 1
MEF2C: Myocyte enhancer factor 2C
NCBI: National Center for Biotechnology Information
NUDT1: Nudix hydrolase 1
NEBL: Nebulette
NHA: Normal human astrocyte
PAX5: Paired box 5
Pers.dis.: Personality disorders
QC: Quality control
Ref: Reference
RNF8: Ring finger protein 8
SIN3A: SIN3 transcription regulator family member A
SNP: Single nucleotide polymorphism
SKA2: Spindle and KT associated 2
SNORA114: Small nucleolar RNA, H/ACA Box 114
SNX8: Sorting nexin 8
SP4: Sp4 transcription factor
SCAS: Spence Children's Anxiety Scale
SUAS: Suicide Assessment Scale
TAL1: T cell acute lymphocytic leukemia protein 1
TFAMP1: Transcription factor A, mitochondrial pseudogene 1
TPR: Translocated promoter region, nuclear basket protein
TTK: TTK protein kinase
UCSC: University of California, Santa Cruz
WMH: World Mental Health
ZW10: Zw10 kinetochore protein
