# Supplementary figures and images for: Methylation in MAD1L1 is associated with the severity of suicide attempt and phenotypes of depression
Source: Clin Epigenetics. 2023 Jan 4;15:1. doi: 10.1186/s13148-022-01394-5 (PMC9811786; doi:10.1186/s13148-022-01394-5)

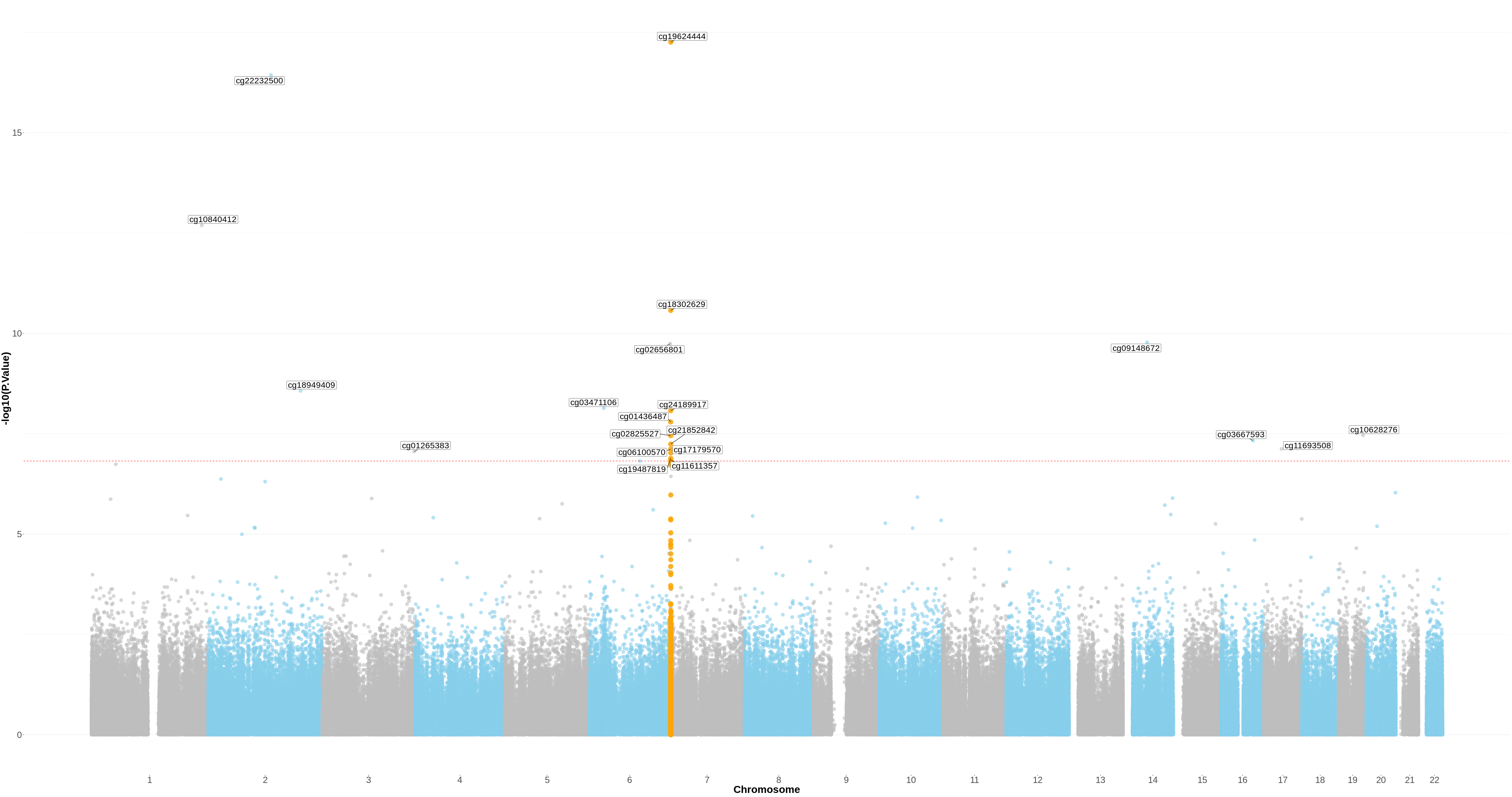

Supplement: Supplementary file 1 — Additional file 1: Fig S1. This Manhattan plot shows identified SNP-related CpG sites in the recall adolescent cohort. This figure comprises results for all investigated SNP sites. All four studied SNPs were tested independently. Analyses were conducted with the R package “limma,” the dominant model was used. The smallest p value for every SNP–CpG association is included in the image. Raw p values from limma are visualized. The dashed red line shows a threshold for significance after correction for multiple testing (false discovery rate) and the number of SNP sites. Only probes that passed the initial QC were included in the analysis. CpG sites located at the MAD1L1 gene are highlighted in yellow. All statistically significant results are annotated by name. Abbreviations: SNP, single nucleotide polymorphism; QC, quality control. [file 13148_2022_1394_MOESM1_ESM.png]

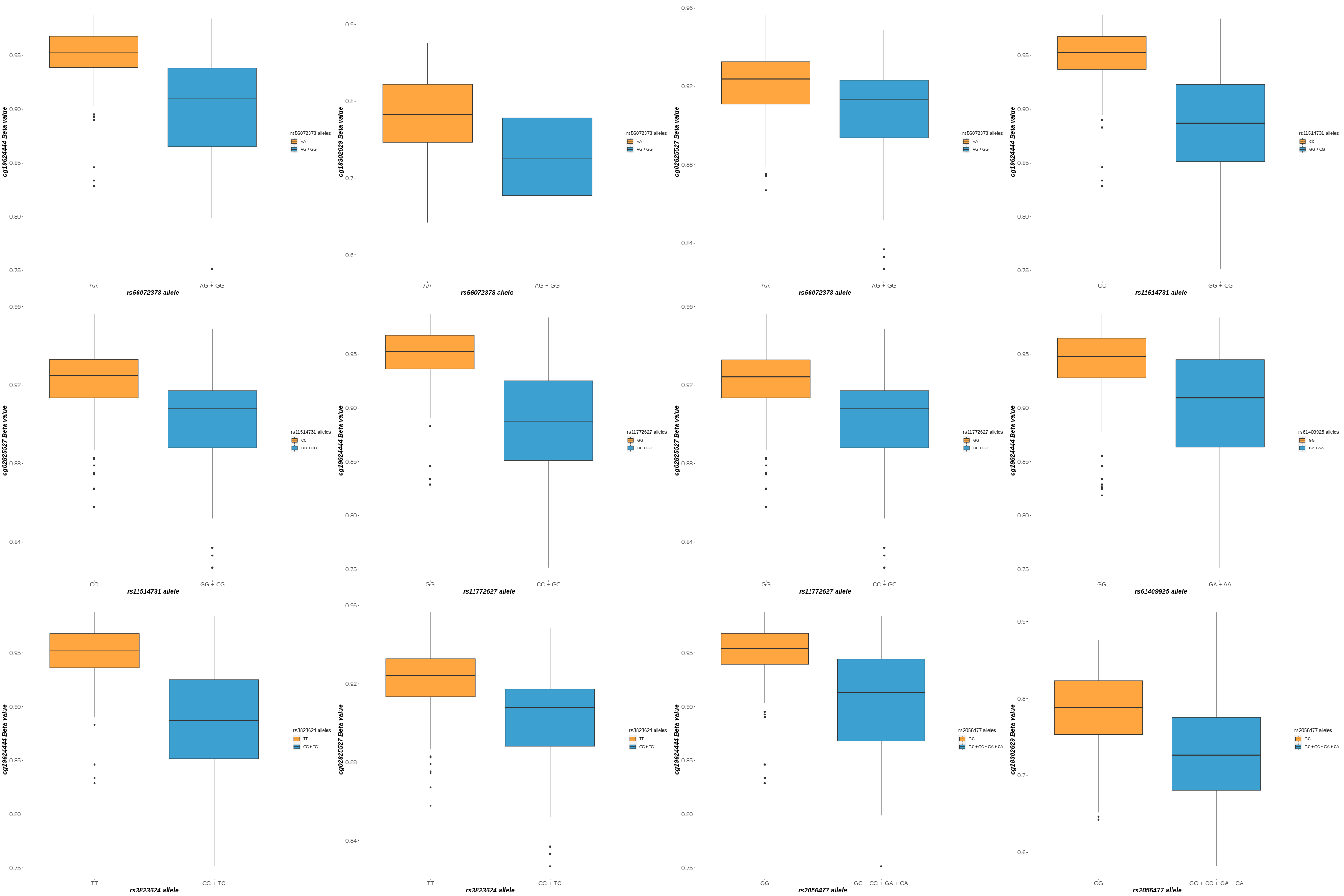

Supplement: Supplementary file 2 — Additional file 2: Fig S2. This figure shows the DNA methylation (β value) of identified lead CpGs in relation to alleles of all related investigated SNPs based on data at screening. All depicted associations were found to be statistically significant after correction for multiple testing and the number of SNP sites using limma-based models. For rs2056477, there were 2 alternative alleles available, and all discovered different genotypes are depicted. [file 13148_2022_1394_MOESM2_ESM.png]

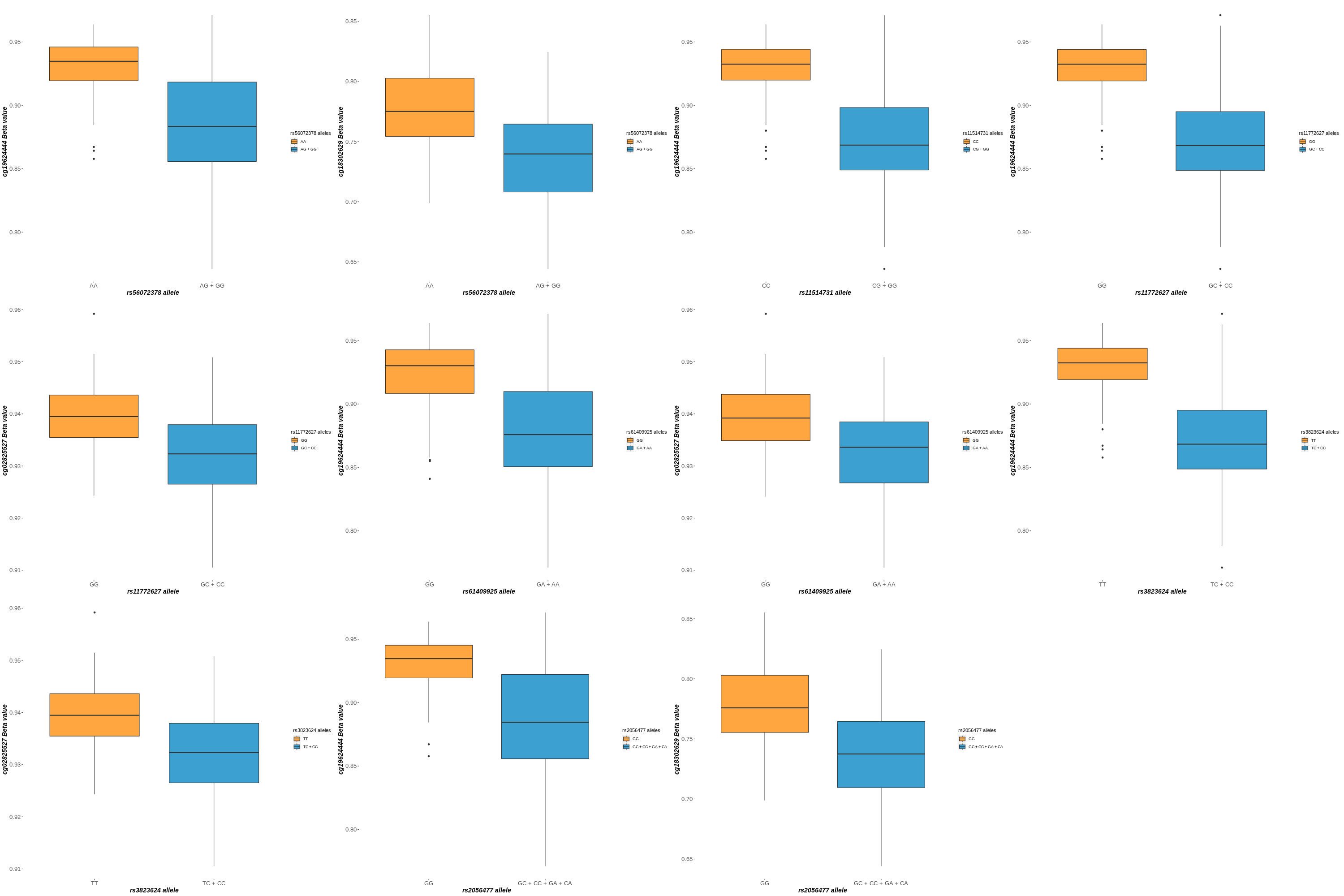

Supplement: Supplementary file 3 — Additional file 3: Fig S3. This figure shows the DNA methylation (β value) of identified lead CpGs in relation to alleles of all related investigated SNPs based on data at recall. All depicted associations were found to be statistically significant after correction for multiple testing and the number of SNP sites. For rs2056477, there were 2 alternative alleles available, and all discovered different genotypes are depicted. [file 13148_2022_1394_MOESM3_ESM.png]

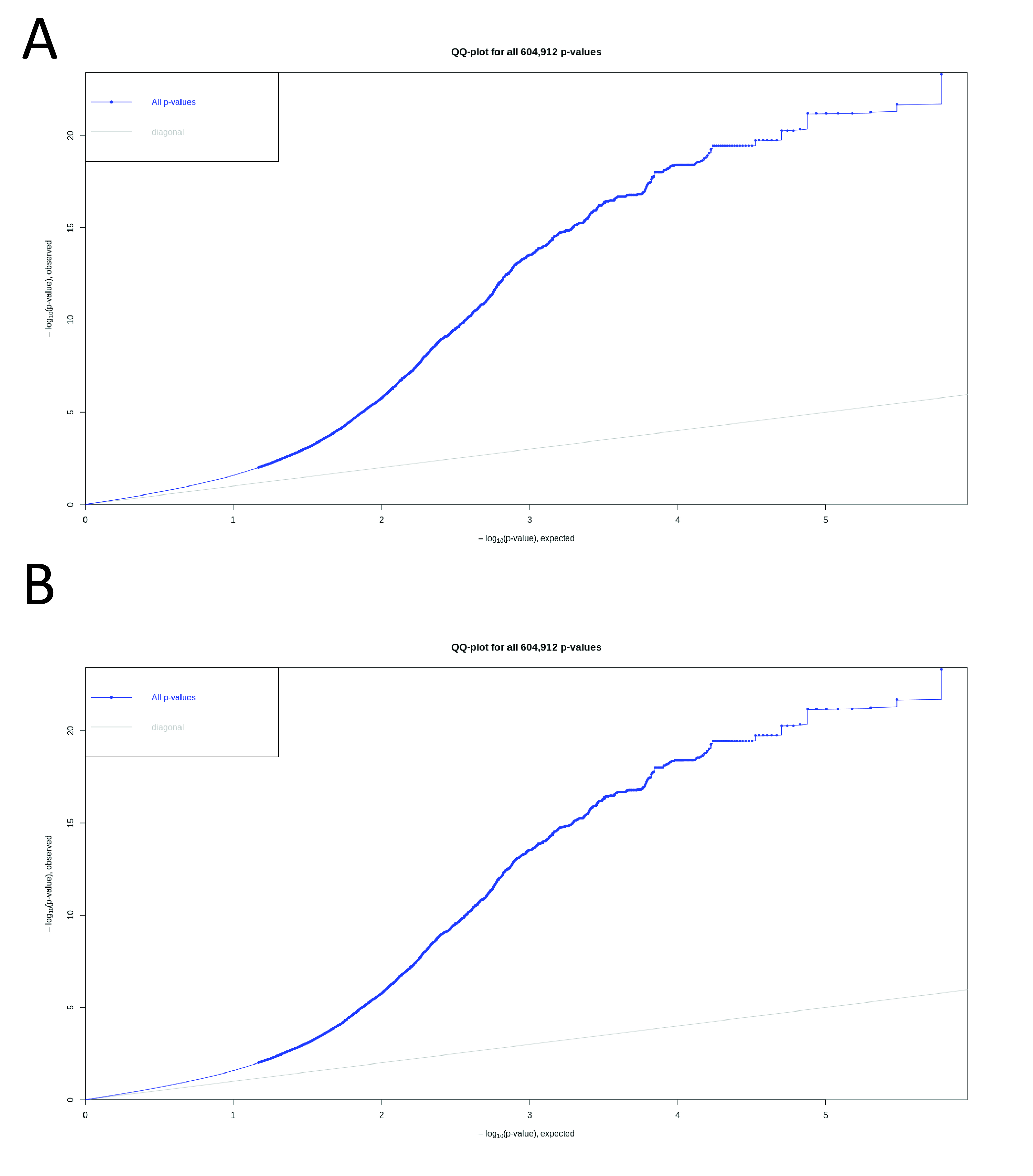

Supplement: Supplementary file 4 — Additional file 4: Fig S4. This figure shows QQ plots of the p-values obtained in the mQTL analysis in the adolescent screening and recall samples. The figure A corresponds to screening, whereas the figure B corresponds to recall. [file 13148_2022_1394_MOESM4_ESM.jpg]

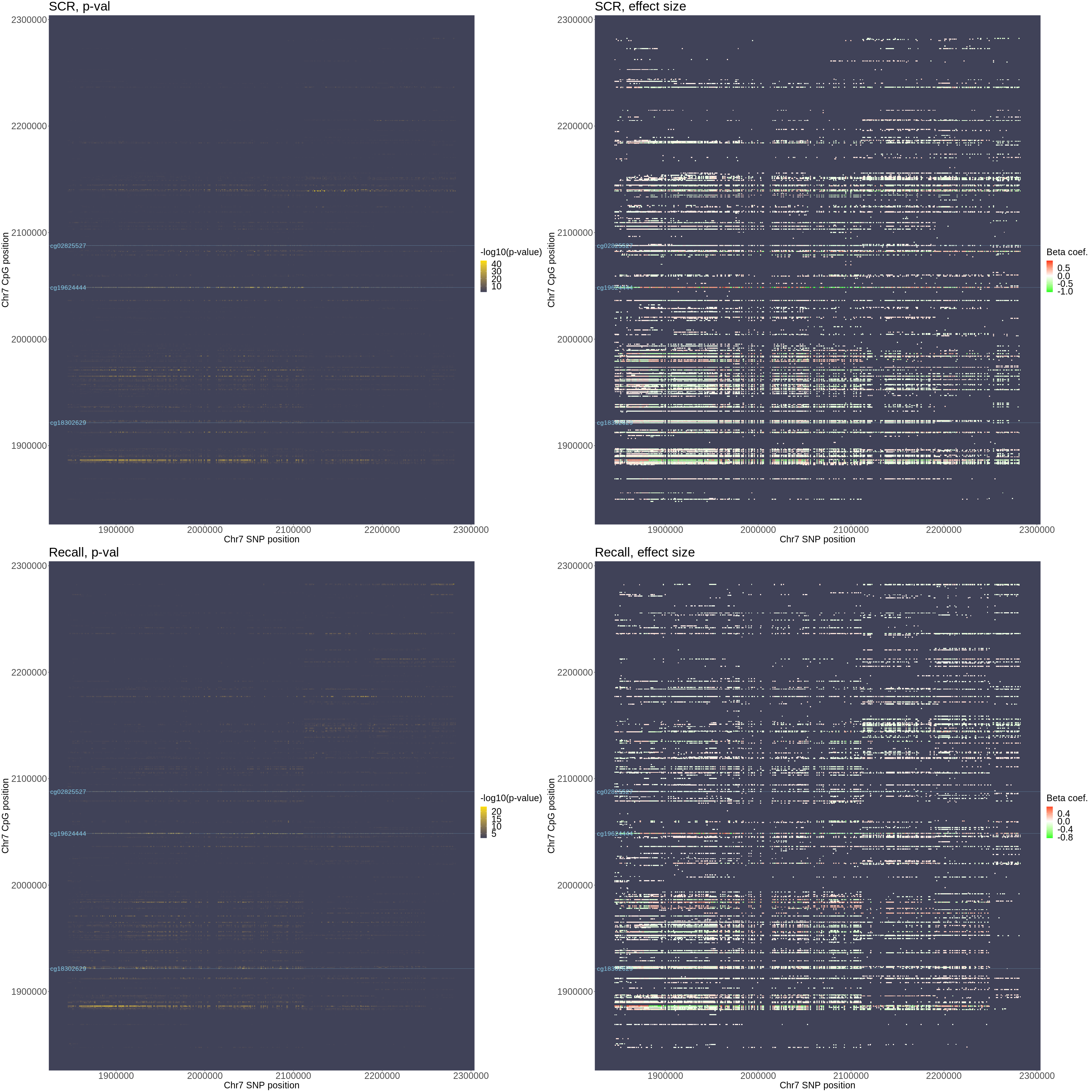

Supplement: Supplementary file 5 — Additional file 5: Fig S5. This figure shows heatmaps of the SNP–CpG interactions in the mQTL analysis. The X-axis shows coordinates for SNPs, the Y-axis represents coordinates for CpGs. Both coordinates correspond to the genome assembly hg19. The first row with plots shows heatmaps for -log10 p values (raw) and beta coefficients in the linear models (adjusted for age, sex, batch, and BMI) for the adolescent screening sample. The second row depicts similar maps for the adolescent recall sample. The linear models were generated with the R package MatrixEQTL and assumed additive genetic effects and the heatmaps were created with ggplot2 (see methods). [file 13148_2022_1394_MOESM5_ESM.png]

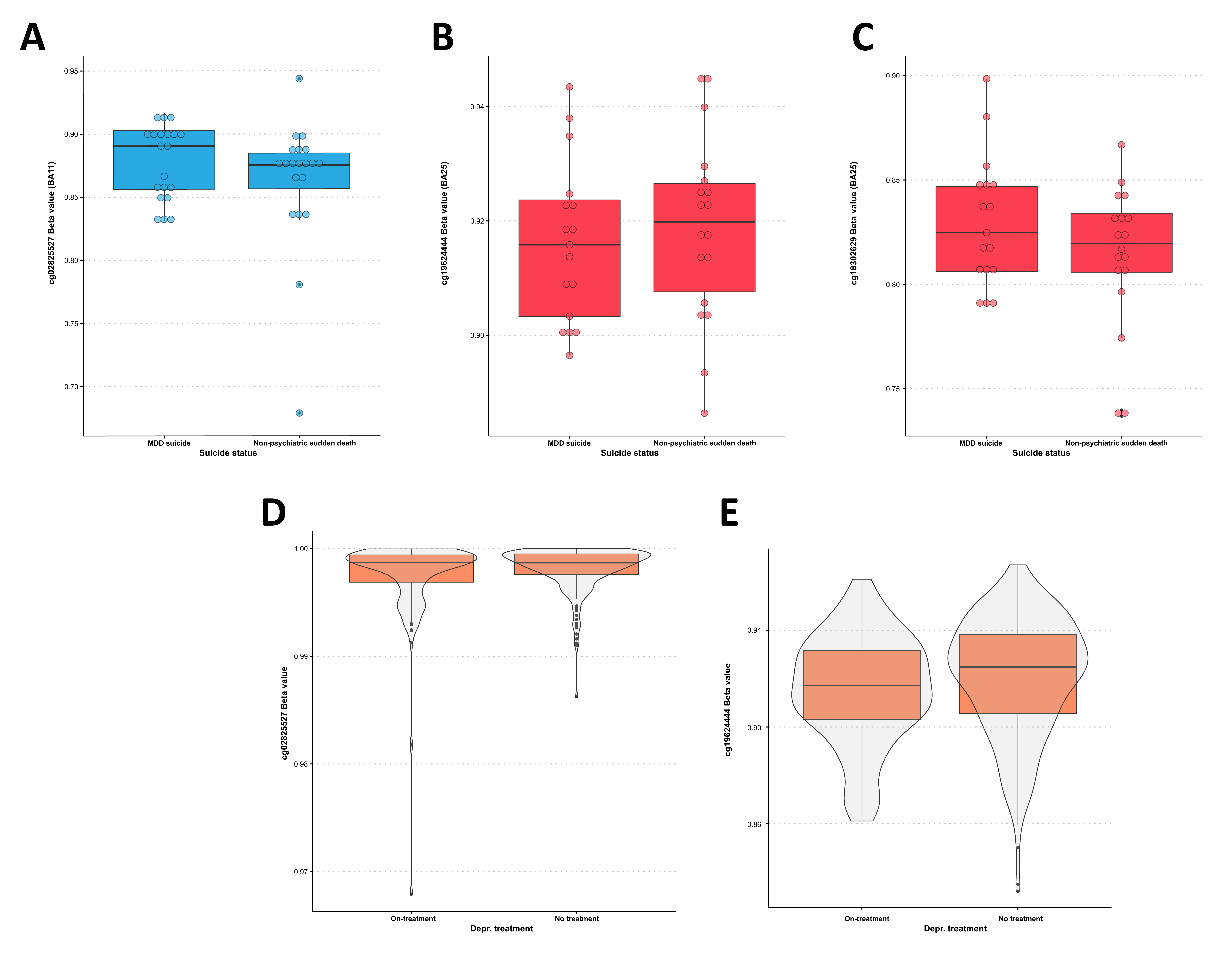

Supplement: Supplementary file 6 — Additional file 6: Fig S6. A. Methylation of cg02825527 in BA11 brain region in the GSE88890 cohort. This figure shows methylation of cg02825527 (β-value) for suicide and control individuals in GSE88890. B. Methylation of cg19624444 in BA25 brain region in the GSE88890 cohort. This figure shows methylation of cg02825527 (β-value) for suicide and control individuals in GSE88890. C. Methylation of cg18302629 in BA25 brain region in the GSE88890 cohort. This figure shows methylation of cg02825527 (β-value) for suicide and control individuals in GSE88890. D. Methylation of cg02825527 in E-GEOD-72680. This graph shows methylation of cg02825527 (β-value) for individuals on antidepressant treatment and without it. E. Methylation of cg19624444 in E-GEOD-72680. This figure depicts methylation of cg19624444 (β-value) for individuals on antidepressant treatment and without it. [file 13148_2022_1394_MOESM6_ESM.jpg]

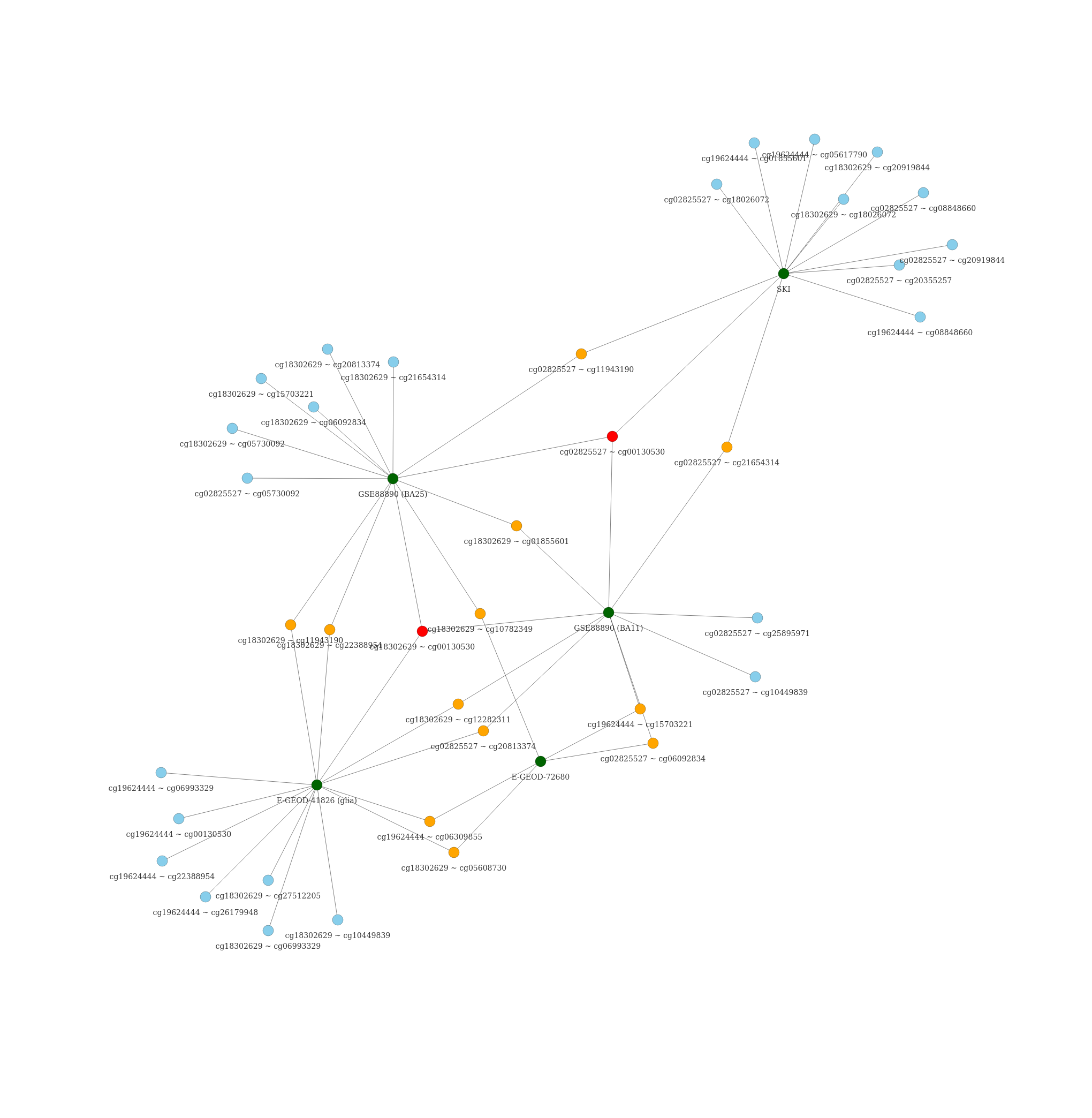

Supplement: Supplementary file 7 — Additional file 7: Fig S7. This figure shows associations between candidate CpGs and stress-related CpGs in the samples where we observed associations (or trends) between MAD1L1 methylation and depression/suicide phenotype. The figure includes all pairs (without accounting for direction) that were nominally significant based on the linear model adjusted for the cohort-specific covariates. The color of the nodes indicates the following: green—sample, blue—CpG–CpG pairs that were observed in one cohort, orange—CpG–CpG pairs that were observed in two cohorts, red—CpG–CpG pairs that were observed in more than two cohorts. [file 13148_2022_1394_MOESM7_ESM.png]

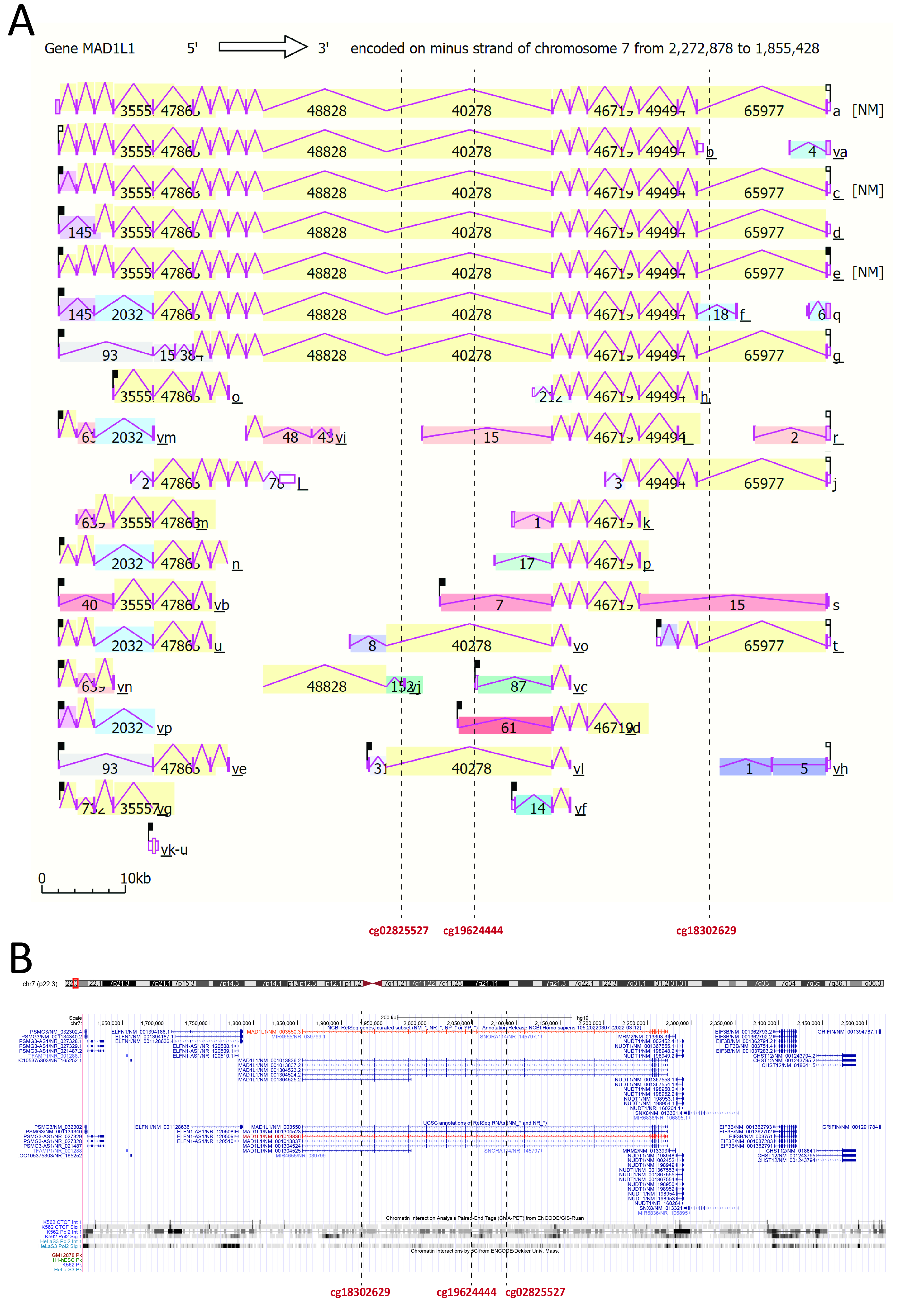

Supplement: Supplementary file 8 — Additional file 8: Fig S8. A. This figure shows alternative transcripts of MAD1L1 obtained from the AceView portal (https://www.ncbi.nlm.nih.gov/IEB/Research/Acembly/av.cgi?db=human&term=MAD1L1&submit=Go) and the relative positions of candidate CpG sites. B. This figure shows NCBI RefSeq tracks of the MAD1L1 gene obtained from the UCSC genome browser. The red MAD1L1 transcript is associated with Illumina HumanHT-12 V4.0 MAD1L1 probes. The orange MAD1L1 transcript is associated with the Affymetrix Human Gene 1.1 ST MAD1L1 probe. [file 13148_2022_1394_MOESM8_ESM.jpg]
