# Supplementary material for: Methylation in MAD1L1 is associated with the severity of suicide attempt and phenotypes of depression
Source: Clin Epigenetics. 2023 Jan 4;15:1. doi: 10.1186/s13148-022-01394-5 (PMC9811786; doi:10.1186/s13148-022-01394-5)
Supplement: Supplementary file 9 — Additional file 9: Table S1. Demographical characteristics of the GSE88890 cohort for two brain regions BA11. Participants were grouped based on the death cause: suicide or non-psychiatric sudden death. For the age variable, sample mean ± standard deviation is shown. Min stands for a minimal value, and max shows the maximal value. [file 13148_2022_1394_MOESM9_ESM.docx]

**Supplementary Table 4. Characteristics of the cohort GSE88890 for two brain regions**

**BA11**

|  | **Study groups** | |
| --- | --- | --- |
|  | **MDD suicide case** | **Non-psychiatric sudden death** |
| Participants | MDD suicide case (n=20, 50%) | Non-psychiatric sudden death (n=20, 50%) |
| Gender distribution | Women: 5 (25%) Men: 15 (75%) | Women: 4 (20%) Men: 16 (80%) |
| Age | 48.6 ± 20.76 Min: 18, Max: 90 | 39.35 ± 19.46 Min: 18, Max: 78 |

**BA25**

|  | **Study groups** | |
| --- | --- | --- |
|  | **MDD suicide case** | **Non-psychiatric sudden death** |
| Participants | MDD suicide case (n=17, 48.57%) | Non-psychiatric sudden death (n=18, 51.43%) |
| Gender distribution | Women: 4 (23.5%) Men: 13 (76.5%) | Women: 4 (22.2%) Men: 14 (77.8%) |
| Age | 49.47 ± 22.44 Min: 18, Max: 90 | 41.22 ± 19.61 Min: 18, Max: 78 |
