# Supplementary material for: Methylation in MAD1L1 is associated with the severity of suicide attempt and phenotypes of depression
Source: Clin Epigenetics. 2023 Jan 4;15:1. doi: 10.1186/s13148-022-01394-5 (PMC9811786; doi:10.1186/s13148-022-01394-5)
Supplement: Supplementary file 10 — Additional file 10: Table S2. Demographical characteristics of the E-GEOD-72680 cohort. Participants were grouped based on the depression treatment status. For numerical variables, sample mean ± standard deviation is shown. Min stands for a minimal value, and max shows the maximal value. Abbreviations: BMI, body mass index; BD, bipolar disorder; CTQ, Childhood Trauma Questionnaire; PTSD, post-traumatic stress disorder; BDI, Beck's Depression Inventory. [file 13148_2022_1394_MOESM10_ESM.docx]

**Supplementary Table 5. Characteristics of the cohort E-GEOD-72680**

|  | **Study groups** | |
| --- | --- | --- |
| **Treatment for depression** | **On-treatment** | **No treatment** |
| Participants | On-treatment (n=137, 36.34%) | No treatment (n=240, 63.66%) |
| Gender distribution | Female: 101 (73.7%) Male: 36 (26.3%) | Female: 164 (68.3%) Male: 76 (31.7%) |
| Age | 44.53 ± 9.93 Min: 20, Max: 70 | 40.05 ± 13.88 Min: 18, Max: 77 |
| BMI | 33.09 ± 7.71 Min: 17.79, Max: 54.97 | 31.99 ± 8.63 Min: 18.22, Max: 72.97 |
| Ethnicity | African American: 117 (85.4%) Caucasian: 17 (12.4%) Mixed: 2 (1.5%) Other: 1 (0.7%) | African American: 234 (97.5%) Caucasian: 5 (2.1%) Mixed: 1 (0.4%) Other: 0 (0%) |
| **Treatment information** | | |
| BD treatment | On-treatment: 29 (21.2%) Without treatment: 95 (69.3%) Unspecified: 13 (9.5%) | On-treatment: 3 (1.2%) Without treatment: 221 (92.1%) Unspecified: 16 (6.7%) |
| PTSD treatment | On-treatment: 36 (26.3%) Without treatment: 99 (72.3%) Unspecified: 2 (1.5%) | On-treatment: 3 (1.2%) Without treatment: 233 (97.1%) Unspecified: 4 (1.7%) |
| Anxiety treatment | On-treatment: 49 (35.8%) Without treatment: 74 (54%) Unspecified: 14 (10.2%) | On-treatment: 7 (2.9%) Without treatment: 217 (90.4%) Unspecified: 16 (6.7%) |
| **Psychiatric scores** | | |
| CTQ total score | 49.53 ± 21.02 Missing val: 4 (2.92%) Min: 25, Max: 119 | 39.49 ± 15.16 Missing val: 4 (1.67%) Min: 25, Max: 102 |
| PTSD symptom scale | 20.76 ± 13.51 Missing val: 7 (5.11%) Min: 0, Max: 47 | 12.48 ± 11.98 Missing val: 7 (2.92%) Min: 0, Max: 50 |
| BDI total score | 20.47 ± 13.95 Missing val: 13 (9.49%) Min: 0, Max: 58 | 14.56 ± 11.56 Missing val: 23 (9.58%) Min: 0, Max: 56 |
| **Life stress scores** | | |
| Cumulative life stress score | 12.49 ± 4 Missing val: 26 (18.98%) Min: 4, Max: 21 | 10.49 ± 3.73 Missing val: 54 (22.5%) Min: 3, Max: 21 |
| Personal life stress score | 8.66 ± 2.96 Missing val: 25 (18.25%) Min: 1, Max: 14 | 7.16 ± 2.81 Missing val: 47 (19.58%) Min: 1, Max: 15 |
| **BDI-categories** | | |
| BDI full categorization | Normal: 39 (28.5%) Mild.mood.dist: 12 (8.8%) Borderline.clin.depr: 13 (9.5%) Mod.depr: 33 (24.1%) Sev.depr: 16 (11.7%) Extr.depr: 11 (8%) Unspecified: 13 (9.5%) | Normal: 94 (39.2%) Mild.mood.dist: 44 (18.3%) Borderline.clin.depr: 23 (9.6%) Mod.depr: 35 (14.6%) Sev.depr: 13 (5.4%) Extr.depr: 8 (3.3%) Unspecified: 23 (9.6%) |
| BDI standard (>=19) | Depressed 67 (48.9%) Non-depressed 57 (41.6%) Unspecified: 13 (9.5%) | Depressed 70 (29.2%) Non-depressed 147 (61.3%) Unspecified: 23 (9.6%) |
| **Kreek-McHugh-Schluger-Kellogg scale for substance abuse** | | |
| Alcohol use | 2.6 ± 3.35 Missing val: 51 (37.23%) Min: 0, Max: 11 | 2.86 ± 3.68 Missing val: 80 (33.33%) Min: 0, Max: 12 |
| Tobacco use | 3.6 ± 3.87 Missing val: 52 (37.96%) Min: 0, Max: 12 | 3.03 ± 3.84 Missing val: 80 (33.33%) Min: 0, Max: 12 |
| Cocaine use | 0.38 ± 1.75 Missing val: 52 (37.96%) Min: 0, Max: 9 | 0.24 ± 1.28 Missing val: 81 (33.75%) Min: 0, Max: 10 |
| Heroin use | 0.35 ± 2.01 Missing val: 53 (38.69%) Min: 0, Max: 13 | 0.05 ± 0.46 Missing val: 83 (34.58%) Min: 0, Max: 5 |
| Marijuana use | 1.2 ± 2.95 Missing val: 52 (37.96%) Min: 0, Max: 12 | 1.7 ± 3.47 Missing val: 82 (34.17%) Min: 0, Max: 12 |
